# Evaluation of the enantioselectivity of new chiral ligands based on imidazolidin-4-one derivatives

**DOI:** 10.3762/bjoc.20.62

**Published:** 2024-04-02

**Authors:** Jan Bartáček, Karel Chlumský, Jan Mrkvička, Lucie Paloušová, Miloš Sedlák, Pavel Drabina

**Affiliations:** 1 Institute of Organic Chemistry and Technology, Faculty of Chemical Technology, University of Pardubice, Studentská 573, 532 10 Pardubice, Czech Republic

**Keywords:** asymmetric aldol reaction, asymmetric Henry reaction, chiral ligands, enantioselective catalysis, imidazolidine derivatives

## Abstract

The new chiral ligands **I–III** based on derivatives of imidazolidin-4-one were synthesised and characterised. The catalytic activity and enantioselectivity of their corresponding copper(II) complexes were studied in asymmetric Henry reactions. It was found that the enantioselectivity of these catalysts is overall very high and depends on the relative configuration of the ligand used; *cis-*configuration of ligand affords the nitroaldols with major enantiomer *S-* (up to 97% ee), whereas the application of ligands with *trans*-configuration led to nitroaldols with major *R*-enantiomer (up to 96% ee). The “proline-type” ligand **IV** was also tested in asymmetric aldol reactions. Under the optimised reaction conditions, aldol products with enantioselectivities of up to 91% ee were obtained.

## Introduction

**Scheme 1:**
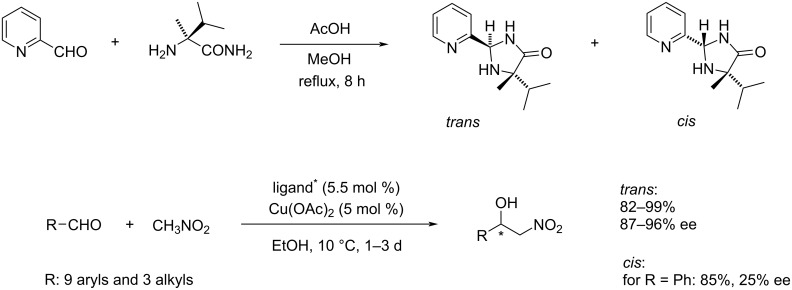
The preparation of 5-isopropyl-5-methyl-2-(pyridin-2-yl)imidazolidin-4-one derivatives and their application in asymmetric Henry reaction [5].

The application of chiral metal complexes as enantioselective catalysts is among the fundamental strategies for preparing compounds in non-racemic forms [1–4]. These complexes typically comprise a chelating chiral ligand capable of coordinating with a metal ion; otherwise, a metal atom itself constitutes a stereocentre [4]. The specific pairing of a chiral ligand and a metal ion is essential for the catalytic characteristics and its effectiveness of the complex in asymmetric syntheses [1–3]. In recent years, our research group has synthesised a series of chiral ligands based on 2-(pyridin-2-yl)imidazolidin-4-one, differentiated by various substitutions at the imidazolidine ring [5–7]. Their copper(II) complexes were evaluated as efficient enantioselective catalysts, particularly in asymmetric Henry reactions (Scheme 1). Subsequent research has led to the development of various catalytic arrangements of the most enantioselective catalysts, including anchoring on polystyrene beads [8–9], magnetic nanoparticles [10], or block copolymers composed of PEG-poly(Glu) [11]. These modifications have facilitated the recycling of the catalysts, offering numerous advantages, including enhanced economic efficiency, eco-friendly practices, and reduced waste. Catalysts with the highest enantioselectivity have been employed in the synthesis of essential chiral intermediates for drug production, such as amprenavir [12], rivaroxaban [13–14], linezolid [13] and salmeterol [7]. Therefore, chiral 2-(pyridin-2-yl)imidazolidin-4-one derivatives stand out as a prominent class of chiral nitrogen ligands in enantioselective catalysis.

**Figure 1:**
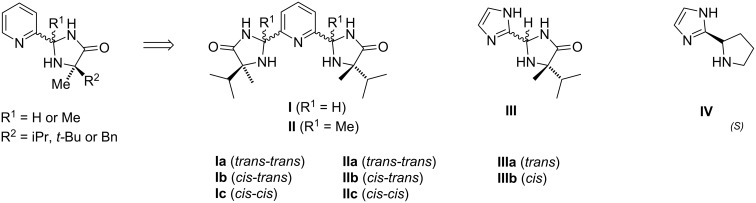
The structure of newly designed ligands **I–IV**.

In this work, we focused on structurally modifying 2-(pyridin-2-yl)imidazolidin-4-one ligands to potentially expand the range of efficient catalysts. Our goal was to synthesise ligands featuring two chiral imidazolidin-4-one units linked to the pyridine ring at the 2- and 6- positions (Figure 1 – ligands **I** and **II**). These newly designed ligands represent tridentate entities. Such structure modification provides the ligands structurally conformable to well-known tridentate chiral ligands: PyBOX [15] and PyBidine [16]. Additionally, we explored further modifications by substituting the pyridine moiety with an imidazole ring in ligand **III** (Figure 1), motivated by DFT calculations (see Supporting Information File 1, part S4) which confirmed that imidazole has a similar donor ability to pyridine. This change reduces the ring size within the ligand structure, potentially increasing the space available for coordinating reacting species, thereby possibly enhancing catalytic activity and enantioselectivity. We also aimed to assess the impact of alkyl groups at the 5-position of the imidazolidine ring on the enantioselectivity of the reaction. Hence, we developed a ligand incorporating an unsubstituted pyrrolidine ring instead of the imidazolidine unit (Figure 1 – ligand **IV**). This structure characterises a 'proline-type' derivative, enabling its use not only in a chiral metal complex catalyst but also as an enantioselective organocatalyst [17]. Accordingly, its application in enantioselective organocatalysis, particularly in asymmetric reactions through “enamine activation”, warrants further investigation.

## Results and Discussion

The corresponding copper(II) acetate complexes of ligands **I–IV** were screened as enantioselective catalysts for asymmetric Henry reactions. The series of nine different aldehydes (aliphatic, aromatic bearing an electron-withdrawing or an electron-donating group, heteroaromatic) was chosen for these studies. The reaction conditions (i.e., temperature, reaction time, amount of catalyst, solvent) were adopted from the pilot study [5] for relevant comparison of catalyst characteristics.

From Table 1 and Table 2, which summarise results obtained using tridentate ligands **Ia–c** and **IIa–c**, it is evident that the catalytic activity their copper(II) complexes is comparable with analogous complexes of bidentate ligands [5] (for TON and TOF parameters see Supporting Information File 1, part S5). The conversions were very high in almost all cases under set reaction conditions, except for pivalaldehyde. Here, the low conversions could be explained by the sterical demand of the aldehyde (R = *t-*Bu), leading to suppressing its coordination with the complex. The ee values achieved with the complex of ligand **Ia** were variable (29–83%), whereas better enantioselectivity was found for less reactive aldehydes (aliphatic and bearing electron-donating group). The copper complex bearing a ligand with *trans*-*trans* configuration (**Ia**) is less enantioselective than those bearing a bidentate ligand analogue (87–96% ee, see [5]). Interestingly, the introduction of a methyl group at position 2 of the imidazolidin-4-one ring, i.e., ligand **IIa**, led to an improvement of enantioselectivity and the ee values obtained by its copper(II) complex were satisfactory (75–96%). This phenomenon was more distinct in the case of the application complexes of ligands with *cis*-*trans* configuration (**Ib** vs **IIb**). Whilst the complex of **Ib** exhibited only poor enantioselectivity, the other afforded the nitroaldols with ee values of 60–90%. Finally, the complexes of ligands with *cis-cis* configuration (**Ic** and **IIc**) were evaluated. These catalyst are the most efficient catalysts, producing nitroaldols with very high enantiomeric purity (approx. 90–95% ee). This finding contrasts with the results obtained in the previous study [5], where the complex of the analogous bidentate ligand with *cis* configuration was found unsuccessful (≈25% ee; see Supporting Information File 1, part S5).

**Table 1:** Asymmetric Henry reactions of various aldehydes with nitromethane catalysed by copper(II) complexes of ligands **Ia–c**.

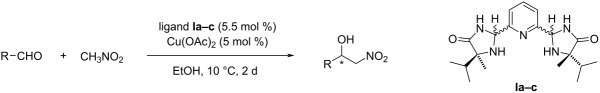						

Aldehyde	Ligand **Ia** (*trans-trans*)		Ligand **Ib** (*cis-trans*)		Ligand **Ic** (*cis-cis*)	
R	Conv^a^ [%]	ee^b^ [%]	Conv^a^ [%]	ee^b^ [%]	Conv^a^ [%]	ee^b^ [%]

Ph	99	60 (*R*)	99	38 (*R*)	99	94 (*S*)
4-NO_2_C_6_H_4_	99	29 (*R*)	99	8 (*R*)	99	91 (*S*)
2-CH_3_OC_6_H_4_	99	80 (*R*)	99	*rac*	99	94 (*S*)
4-ClC_6_H_4_	99	52 (*R*)	99	7 (*R*)	99	95 (*S*)
2-thienyl	94	66 (*R*)	99	20 (*R*)	86	95 (*S*)
naphth-2-yl	96	69 (*R*)	94	25 (*R*)	94	93 (*S*)
PhCH_2_CH_2_	98	73 (*R*)	91	38 (*R*)	95	89 (*S*)
*t-*Bu	35	80 (*R*)	30	41 (*R*)	32	96 (*S*)
iPr	98	83 (*R*)	94	43 (*R*)	99	91 (*S*)

^a^The conversion was determined by ^1^H NMR analysis of the crude product. ^b^The enantiomeric excess was determined by chiral HPLC.

**Table 2:** Asymmetric Henry reactions of various aldehydes with nitromethane catalysed by copper(II) complexes of ligands **IIa–c**.

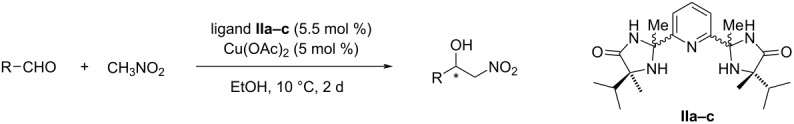						

Aldehyde	Ligand **IIa** (*trans-trans*)		Ligand **IIb** (*cis-trans*)		Ligand **IIc** (*cis-cis*)	
R	Conv^a^ [%]	ee^b^ [%]	Conv^a^ [%]	ee^b^ [%]	Conv^a^ [%]	ee^b^ [%]

Ph	99	80 (*R*)	99	70 (*R*)	99	90 (*S*)
4-NO_2_C_6_H_4_	99	75 (*R*)	99	60 (*R*)	99	82 (*S*)
2-CH_3_OC_6_H_4_	99	96 (*R*)	99	77 (*R*)	99	86 (*S*)
4-ClC_6_H_4_	99	88 (*R*)	99	85 (*R*)	99	96 (*S*)
2-thienyl	95	90 (*R*)	98	86 (*R*)	97	97 (*S*)
naphth-2-yl	97	93 (*R*)	97	86 (*R*)	96	96 (*S*)
PhCH_2_CH_2_	92	75 (*R*)	95	87 (*R*)	97	91 (*S*)
*t-*Bu	31	92 (*R*)	42	93 (*R*)	48	96 (*S*)
iPr	98	96 (*R*)	99	90 (*R*)	93	96 (*S*)

^a^The conversion was determined by ^1^H NMR analysis of the crude product. ^b^The enantiomeric excess was determined by chiral HPLC.

**Figure 2:**
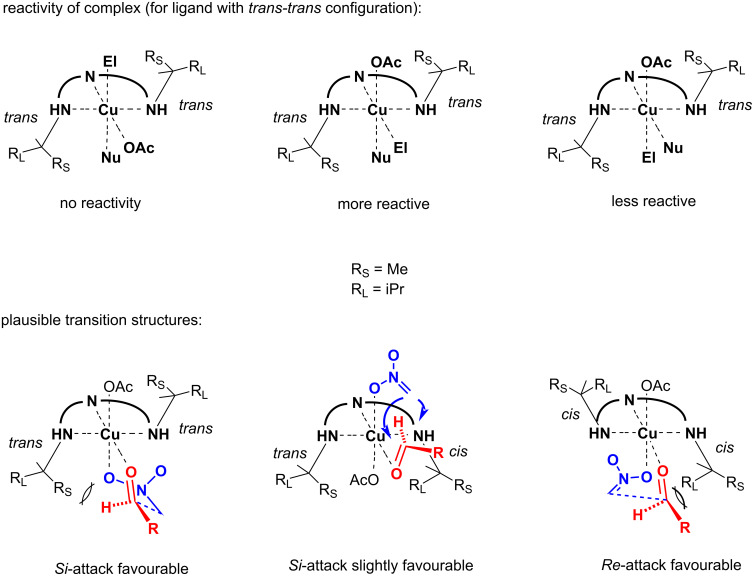
Plausible transition structures for the Henry reaction.

According to expectations resulted from previous studies [5–14], the complexes of the ligands **Ia** and **IIa** afforded the nitroaldols with the prevailing configuration *R*, while the complexes of **Ic** and **IIc** with prevailing configuration *S*. The application of the complexes of the ligands **Ib** and **IIb** (*cis-trans* form) also led in all cases to nitroaldols with some excess of the *R*-enantiomer. A possible explanation of these results is illustrated in Figure 2, which shows plausible transition structures for the Henry reaction. Due to the Jahn–Teller effect, i.e., distortion of the octahedral Cu(II) complex forming four equatorial and two perpendicular coordination sites [18], the effective transition state includes the electrophile positioned in the equatorial site (strongly coordinated) and the nucleophile in the perpendicular site (weakly coordinated) [19]. The most favourable orientation of aldehyde should be out of the ligand’s molecular parts, thus forming *E*-configuration at the C=O bond. In this manner, the asymmetric induction results in the restricted addition of nitromethane influenced by imidazolidin-4-one moieties. The higher enantioselectivity of complexes of the ligands with *cis-cis* configuration could be explained by the fact that the larger (R_L_) alkyl group is located toward the reactants. Thus, it blocks unfavourable *Si*-attack more effectively. Similarly, for the complexes bearing *cis-trans* ligands, *Si*-attack is preferred due to the higher restriction of imidazolidin-4-one with *cis*-configuration, i.e., by more significant steric demand of the R_L_ group.

Next, Table 3 summarises the results of Henry reactions obtained by catalysis with copper(II) complexes of ligands **IIIa**,**b**. The mutual comparison of these catalysts with those composed of analogous 2-(pyridin-2-yl)imidazolidin-4-ones [5] follows several significant conclusions. For instance, the catalyst of ligand **IIIa** possesses lower catalytic activity than the pyridine derivative (for TON and TOF parameters see Supporting Information File 1, part S5). The conversions were poor in the case of less reactive aldehydes, e.g., thiophene-2-carbaldehyde, and 3-phenylpropionaldehyde. To achieve satisfactory yields, the reaction time could be extended. Nevertheless, the ee values obtained by catalysts of **IIIa** vary in the range of 84–96%; hence its enantioselectivity is comparable with the pyridine analogue [5]. Notably, the complex of ligand **IIIb** having *cis*-configuration is more enantioselective than the pyridine derivative [5] (see Supporting Information File 1, part S5); the ee values were overall high (74–92%), however, a little bit lower than those achieved by the complex of ligand **IIIa** (Δ 1–15% ee).

**Table 3:** Asymmetric Henry reactions of various aldehydes with nitromethane catalysed by copper(II) complexes of ligands **IIIa** and **IIIb**.

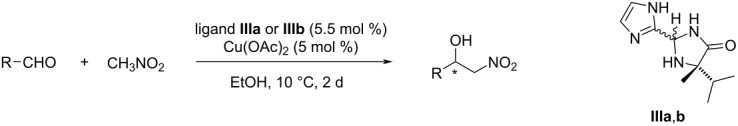				

Aldehyde	Ligand **IIIa** (*trans*)		Ligand **IIIb** (*cis*)	
R	Conv^a^ [%]	ee^b^ [%]	Conv^a^ [%]	ee^b^ [%]

Ph	63	89 (*R*)	57	86 (*S*)
4-NO_2_C_6_H_4_	97	84 (*R*)	89	76 (*S*)
2-CH_3_OC_6_H_4_	98	94 (*R*)	89	93 (*S*)
4-ClC_6_H_4_	82	89 (*R*)	76	85 (*S*)
2-thienyl	40	88 (*R*)	36	80 (*S*)
naphth-2-yl	63	89 (*R*)	63	84 (*S*)
PhCH_2_CH_2_	45	89 (*R*)	44	74 (*S*)
*t-*Bu	62	96 (*R*)	49	92 (*S*)
iPr	90	94 (*R*)	64	88 (*S*)

^a^The conversion was determined by ^1^H NMR analysis of the crude product. ^b^The enantiomeric excess was determined by chiral HPLC.

Further, the copper(II) complex of ligand **IV** were also tested for asymmetric Henry reaction. The ligand **IV** structure arose from the ligand **IIIb** structure – the imidazolidin-4-one ring present at ligand **IIIb** was formally replaced by a pyrrolidine one. Here, we presume the comparable coordinating ability of both species of heterocycles. However, the pyrrolidine cycle of ligand **IV** does not contain alkyl groups at position 5. Therefore, the ee values obtained by the complex of ligand **IV** in the Henry reaction could be used to estimate the impact of this substitution on the resulting enantioselectivity of the studied ligands. The results summarised in Table 4 show that the complex of ligand **IV** is significantly less enantioselective (37–55% ee) than the complex of ligand **IIIb**. Thus, the alkyl substitution at position 5 of the imidazolidin-4-one or pyrrolidine ring of ligands **I–IV** can be considered as a fundamental part of the ligand’s structure, which enables them to possess high enantioselectivity. All nitroaldols were obtained with an excess of *S*-enantiomer, likewise in catalysis by copper(II) complexes of ligands **Ic**, **IIc** and **IIIb,** as well as ligands based on pyridine-(imidazolidin-4-one) [5–7] owned the *S*-configuration at the imidazolidin-4-one ring (see Supporting Information File 1, part S5). Hence, the configuration of a ligand at position 2 determines the type of enantiomer of nitroaldol in excess and the environment around the stereogenic centre at position 5 affects the resulting value of ee.

**Table 4:** Asymmetric Henry reactions of various aldehydes with nitromethane catalysed by copper(II) complex of ligand **IV**.

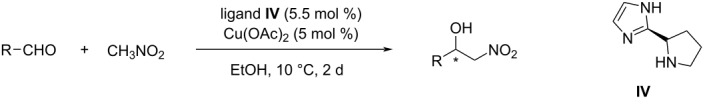		

Aldehyde	Ligand **IV**	
R	Conv^a^ [%]	ee^b^ [%]

Ph	97	50 (*S*)
4-NO_2_C_6_H_4_	99	37 (*S*)
2-CH_3_OC_6_H_4_	99	55 (*S*)
4-ClC_6_H_4_	91	50 (*S*)
2-thienyl	99	36 (*S*)
naphth-2-yl	98	46 (*S*)
PhCH_2_CH_2_	92	50 (*S*)
*t-*Bu	40	52 (*S*)
iPr	75	49 (*S*)

^a^The conversion was determined by ^1^H NMR analysis of the crude product. ^b^The enantiomeric excess was determined by chiral HPLC.

The structure of compound **IV** is similar to the well-known organocatalyst 5-(*S*)-pyrrolidin-2-yl-1*H*-tetrazole, which was successfully used in many asymmetric reactions via “enamine activation”, especially in asymmetric aldol reactions [20–23]. Moreover, compound **IV** was previously included in a study dealing with asymmetric cascade reactions (based on aldol reactions) of aldehydes with α-keto acids producing chiral isotetronic acids. However, the application of compound **IV** as the organocatalyst in these reactions proceeded sluggishly, and the corresponding products were obtained in only moderate ees [24]. Herein, the aldol reaction was chosen as a standard asymmetric reaction to explore the catalytic features of the proline derivate **IV** in detail.

Although various proline derivatives have been described for the use in asymmetric aldol reactions [25], the optimal reaction conditions for achieving maximal enantioselectivity and conversions vary and must be tailored to the specific catalyst used. Thus, early attempts at the aldol reaction of 4-nitrobenzaldehyde with cyclohexanone were performed to evaluate the reaction parameters, i.e., solvent, reaction temperature, and amount of acidic additive (Table 5). Hence, using DMF at −25 °C were the most convenient conditions regarding diastereo- and enantioselectivity and chemical yield. Next, the series of aromatic aldehydes with different substitutions were tested under the set reaction conditions. The yields decreased expectably in the range from aldehydes with electron-withdrawing substituents to that with electron-donating substituents. Thus, 4-tolualdehyde afforded the aldol product only with a moderate yield of 30%; however, the diastereo- and enantioselectivity was satisfactory. A similar result was obtained in the reaction of 4-nitrobenzaldehyde with acetone. The comparison of **IV** with 5-(*S*)-pyrrolidin-2-yl-1*H*-tetrazole shows that both proline derivatives possess similar enantioselectivity. However, compound **IV** is less catalytically active, probably due to the lower acidity of protonated 1*H*-imidazole than 1*H*-tetrazole [17].

**Table 5:** Asymmetric aldol reactions of various aldehydes with ketones catalysed by compound **IV**.

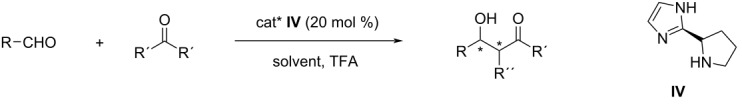								

Aldehyde R	Ketone R´-CO-R´	Temp [°C]	Solvent	TFA [mol %]	Time [d]	Yield [%]	dr*^a^* (*anti*/*syn*)	ee^b^* (anti)* [%]

4-NO_2_C_6_H_4_	-(CH_2_)_5_-	20	MeOH	20	5	88	2.25:1	73
4-NO_2_C_6_H_4_	-(CH_2_)_5_-	3	MeOH	20	5	82	2.70:1	78
4-NO_2_C_6_H_4_	-(CH_2_)_5_-	3	MeOH	40	5	89	2.60:1	82
4-NO_2_C_6_H_4_	-(CH_2_)_5_-	3	DMF	20	3	96	2.70:1	83
4-NO_2_C_6_H_4_	-(CH_2_)_5_-	–25	DMF	20	5	82	7.70:1	90
4-NO_2_C_6_H_4_	-(CH_2_)_5_-	–25	DMF	40	5	95	20.0:1	89
4-NO_2_C_6_H_4_	-(CH_2_)_5_-	–25	DMSO	40	5	40	2.50:1	81
4-NO_2_C_6_H_4_	-(CH_2_)_5_-	–25	MeCN	40	14	26	6.70:1	91
4-CNC_6_H_4_	-(CH_2_)_5_-	–25	DMF	40	5	85	2.35:1	85
4-ClC_6_H_4_	-(CH_2_)_5_-	–25	DMF	40	5	88	4.00:1	82
Ph	-(CH_2_)_5_-	–25	DMF	40	5	56	11.0:1	86
4-CH_3_C_6_H_4_	-(CH_2_)_5_-	–25	DMF	40	5	30	20.0:1	85
4-NO_2_C_6_H_4_	CH_3_-	–25	DMF	40	5	36	–	84

^a^The dr was determined by ^1^H NMR analysis of the crude product. ^b^The enantiomeric excess was determined by chiral HPLC.

## Conclusion

In this study, we successfully synthesised enantiomerically pure stereoisomers of 2,2'-(pyridin-2,6-diyl)-bis(imidazolidin-4-one) derivatives, **Ia–c** and **IIa–c**, analogous to well-known tridentate chiral ligands such as PyBOX [15] and PyBidine [16]. The copper(II) complexes of these ligands demonstrated remarkable enantioselectivity in asymmetric Henry reactions, achieving enantiomeric excess values exceeding 90% in numerous instances, categorising them among the most efficient catalysts for this reaction type. A key finding of our research was the identification of position 2 on the imidazolidine-4-one unit as the crucial site for asymmetric induction. While the nature of the substituent at this chiral centre (i.e., hydrogen or methyl) influenced the catalytic results, it was always part of a more complex interplay involving the ligand–substrate combination, suggesting that the type of the substituent was not the sole determining factor.

Our investigation also revealed that the configuration of the chiral centre at position 5 significantly influences the catalyst's activity. While the bidentate 2-(pyridin-2-yl)imidazolidin-4-one ligands with a *trans* configuration at this position outperformed their *cis* counterparts (see Supporting Information File 1, part S5), the tridentate ligands (**Ia–c** and **IIa–c**) exhibited higher enantioselectivity with a *cis* configuration at position 5 relative to position 2. Additionally, when mixed configurations were present at position 2 in tridentate ligands (**Ib** and **IIb**), the 2*R* configuration facilitated more effective chirality transfer.

Substituting the pyridine moiety with an imidazole in ligands **IIIa**,**b** led to a lower reaction rate compared to their pyridine counterparts. However, the *cis* diastereomer (**IIIb**) showed significantly enhanced enantioselectivity compared to its equivalent pyridine-based ligand with the same configuration (see Supporting Information File 1, part S5). This change from pyridine to imidazole lowered the reaction rate but did not alter the critical role of configuration at position 2 in determining the enantiomeric purity of the final product.

Furthermore, the study of “proline-type” derivative **IV** underscored the importance of position 2 in chirality transfer, although with reduced efficiency in the absence of a second stereogenic centre at position 5. Nonetheless, **IV** proved to be an effective enantioselective organocatalyst in the asymmetric aldol reaction, matching the enantioselectivity levels of other proline-heterocycle derivatives [25].

Overall, our findings underscore the paramount importance of the stereochemistry at position 2 in chiral ligands dictating enantioselectivity. This study provides essential insights for the design of asymmetric catalysts, especially for Henry and aldol reactions.

## Supporting Information

File 1 General information and experimental data of prepared compounds, copies of ^1^H and ^13^C NMR spectra and DFT calculations.

## Data Availability

All data that supports the findings of this study is available in the published article and/or the supporting information to this article.
