# Supplementary material for: Evaluation of the enantioselectivity of new chiral ligands based on imidazolidin-4-one derivatives
Source: Beilstein J Org Chem. 2024 Apr 2;20:684–91. doi: 10.3762/bjoc.20.62 (PMC10999975; doi:10.3762/bjoc.20.62)
Supplement: File 1 — General information and experimental data of prepared compounds, copies of 1H and 13C NMR spectra and DFT calculations. [file Beilstein_J_Org_Chem-20-684-s001.pdf]

BEILSTEIN JOURNAL OF ORGANIC CHEMISTRY

Supporting Information

for

Evaluation of the enantioselectivity of new chiral ligands based on imidazolidin-4-one derivatives

Jan Bartáček, Karel Chlumský, Jan Mrkvička, Lucie Paloušová, Miloš Sedlák and Pavel Drabina

Beilstein J. Org. Chem. **2024,** *20,* 684–691. doi:10.3762/bjoc.20.62

General information and experimental data of prepared compounds, copies of ^{1}H and ^{13}C NMR spectra and DFT calculations

License and Terms: This is a supporting information file under the terms of the Creative Commons Attribution License (https://creativecommons.org/licenses/by/4.0). Please note that the reuse, redistribution and reproduction in particular requires that the author(s) and source are credited and that individual graphics may be subject to special legal provisions.
The license is subject to the *Beilstein Journal of Organic Chemistry* terms and conditions: (https://www.beilstein-journals.org/bjoc/terms)

Table of content

1. Discussion of synthesis of ligands........ S2
2. Experimental procedures........ S5
    2.1. General procedures........ S5
    2.2. Synthesis........ S6
3. NMR spectra........ S13
4. DFT calculations........ S40
5. Summarisation of the results of catalytic experiments........ S45
6. References and notes........ S46

1. Discussion of synthesis of ligands

The 2,2´-(pyridine-2,6-diyl)-bis(imidazolidine-4-one) ligands **Ia–c** were prepared according to the modified protocol described previously (see Scheme 1 in the main text) for the synthesis of analogous bidentate 2-(pyridine-2-yl)imidazolidine-4-one ligand (R^1 = H; R^2 = iPr) [1]. The formation of the ligands **Ia–c** consists of the condensation reaction of pyridine-2,6-dicarbaldehyde with (*S*)-2-amino-2,3-dimethylbutanamide (Scheme 1).

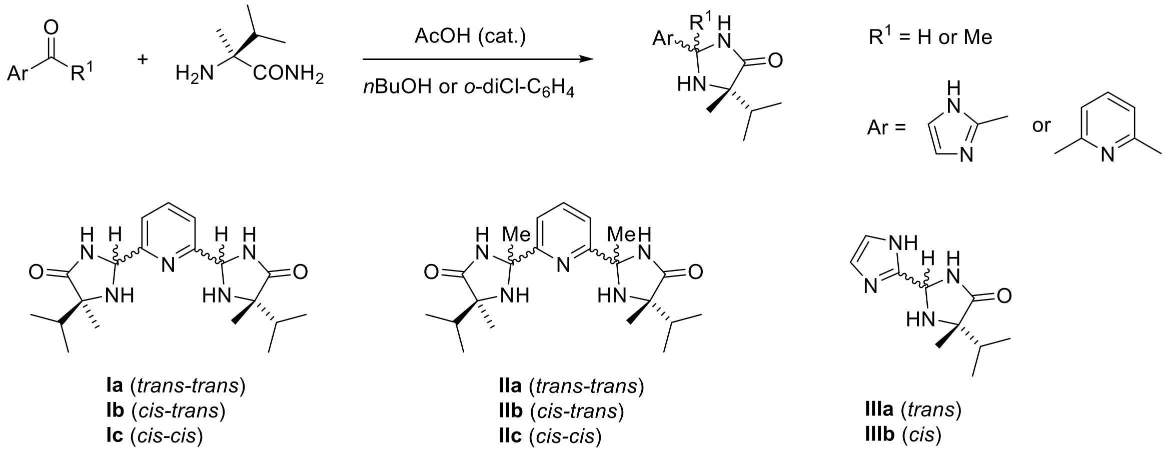

Scheme 1. The synthesis of the ligands **I-III**.

To obtain the corresponding bis(imidazolidine-4-one) derivatives in satisfactory yields and to avoid the appearance of mono(imidazolidine-4-one) derivative intermediates in the crude product mixture, it was necessary to explore the appropriate reaction conditions. Key to the successful synthesis was the use of an excess of the 2-aminoamide reagent (3 equiv; 1.5 equiv to each carbonyl group) and an elevated reaction temperature of 80 °C. For this reason, the initially used solvent (MeOH) [1] needed to be replaced by *n*-BuOH. The reaction time was extended to 120 h, under an inert argon atmosphere, to ensure complete conversion to ligands **Ia-c** while simultaneously preventing the formation of mono(imidazolidine-4-one) intermediates and avoiding any undesirable oxidation to imidazoline-4-one derivative. This approach led to the high-yield production of ligands **Ia–c** (Figure 1), with the diastereomers

being formed in a ratio of 1:2:1 (**Ia**:**Ib**:**Ic**). The final isolation of each diastereomer was achieved by careful column chromatography on silica using a mixture of acetone, AcOEt, and MeOH (13/7/1) as the eluent. Multiple fractionation stages were necessary to effectively separate the mixture into distinct components.

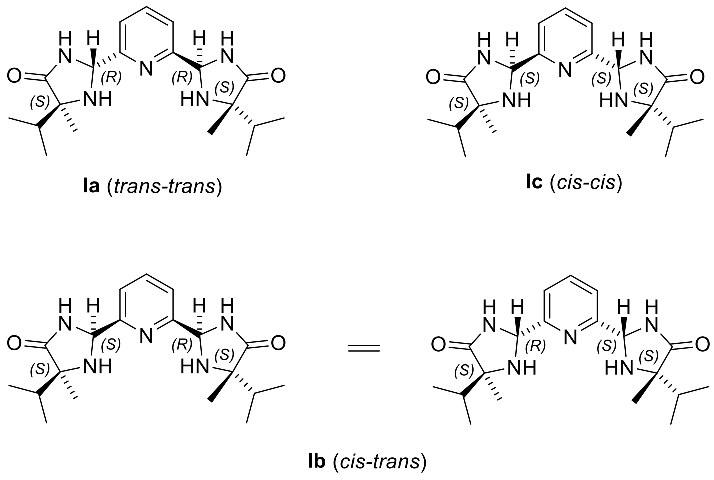

Figure 1 The relative configuration at imidazolidine-4-one rings of ligands **Ia-c**.

Inspired by the methodology outlined in reference [1] for the synthesis of a bidentate ligand (R^1 = Me; R^2 = iPr), and similar to the process used for the preparation of ligands **Ia**–**c**, ligands **IIa**–**c** were synthesised through a reaction between 2,6-diacetylpyridine and (*S*)-2-amino-2,3-dimethylbutanamide (3 equiv). A notable adaptation in this synthesis was the employment of *ortho*-dichlorobenzene as the solvent, enabling the reaction to proceed efficiently at the elevated temperature of 140 °C. TLC was used to monitor the reaction, with the disappearance of mono-(imidazolidine-4-one) derivatives observed after 96 h. The subsequent chromatographic separation (SiO_2; AcOEt/MeOH (20/1) yielded the individual diastereomers *trans-trans* (**IIa**), *cis-trans* (**IIb**), and *cis-cis* (**IIc**) in a ratio of 2:3:1, achieving an overall yield of 70%.

Building upon the methodologies employed for the earlier ligands and following the procedures outlined in reference [1] for synthesising 2-(pyridine-2-yl)imidazolidine-4-one analogues, the

synthesis of ligands **IIIa**,**b** was achieved via a condensation reaction of (*S*)-2-amino-2,3-dimethylbutanamide with 1*H*-imidazole-2-carbaldehyde. Unlike the previous reaction conditions (for **Ia–c**; MeOH; 65 °C; 8 h) that predominantly produced the Schiff base intermediate, the imidazolidine-4-one derivatives **IIIa**,**b** were successfully obtained under modified conditions. This involved refluxing in *n*-BuOH for 17 h, leading to a good yield (66%) of the desired products. The individual epimers **IIIa** and **IIIb** (in the ratio of 3:4) were then effectively separated using column chromatography (SiO_2; acetone), ensuring the purity of the final compounds.

Following the established synthetic pathway detailed in references [2,3], compound **IV** was initially prepared starting from *N*-Boc-prolinol. The process involved synthesising (*S*)-2-(*N*-Boc-pyrrolidine-2-yl)-1*H*-imidazole. However, the deprotection step, traditionally conducted under conditions such as TFA/DCM [2] and $BF_3{\cdot}Et_2O$ [3], resulted in the formation of numerous undesired by-products. To address this issue, we modified the method by replacing the Boc protecting group with a CBz group (Scheme 2). This alteration allowed for the successful oxidation of *N*-CBz-prolinol via Swern oxidation, yielding the corresponding aldehyde with high efficiency (75%) [4]. Subsequently, this aldehyde was converted to the imidazole derivative, following the original protocol, with a yield of 48% [2,3]. The final step involved hydrogenolysis for the deprotection of the pyrrolidine moiety, resulting in the formation of ligand **IV** as a pale-yellow oil. Notably, no racemisation was observed throughout the reaction process.

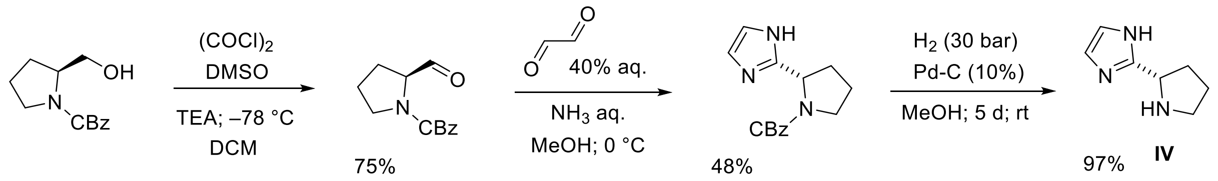

Scheme 2. The synthesis of the ligand **IV**.

2. Experimental procedures

2.1. General procedures

The starting chemicals and solvents were obtained from TCI Chemicals or Fluorochem and used without further purification. (*S*)-2-Amino-2,3-dimethylbutanamide was prepared according to the method described previously [5]. Column chromatography was performed using 60 Å (60–200 μm) silica gel. TLC was performed on aluminium-backed silica gel plates (Merck DC, Alufolien Kieselgel 60 F_{254}) with spots visualised by UV light. The melting point temperatures are uncorrected. The IR spectra were measured at room temperature using Thermo Scientific Nicolet iS50 FT-IR Spectrometer with ATR technique, the resolution was 4 cm^{-1}, FT-IR data are presented in cm^{-1}. ^{1}H NMR spectra were recorded on a Bruker Avance 400 instrument (400.13 MHz for 1H) or Bruker Ascend 500 instrument (500.13 MHz for ^{1}H). Chemical shifts δ were referenced to the residual peak of $CDCl_3$ at 7.26 ppm or MeOD-d_4 at 3.31 ppm. The ^{13}C NMR spectra were calibrated with respect to the middle signal in the triplet of $CDCl_3$ (δ = 77.23 ppm). High-resolution mass spectra were measured on the Thermo Fisher Scientific MALDI LTQ Orbitrap instrument. The used matrix was a 0.2 M solution of 2,5-dihydroxybenzoic acid (DHB) in MeCN/H_2O (95:5). Spectra were calibrated with respect to the used matrix. The optical rotation was measured on a Perkin–Elmer 341 instrument; the concentration *c* was given in g/100 mL. HPLC analyses were performed on the Watrex HPLC instrument with UV-Vis DAD (200–800 nm) SYKAM 3240 and with chiral Daicel columns Chilacel OD-H, Chiralpak AD-H, Chiralpak IA and Chiralpak AS-H (250 mm × 4.6 mm). Hydrogenations were performed in pressure vessel Berghof BR-100. To evaluate the effectiveness of the catalysts, the values of Turnover Number (TON) and Turnover Frequency (TOF) related to the production of the major stereoisomer were calculated using the following equations:

$$TON = \frac{\frac{yield\ (\%)\cdot(\frac{ee\ (\%)}{2}+50)}{100}}{mol\ \%\ cat.}\ ;\ TOF = \frac{TON}{time\ (h)}$$

2.2. Synthesis

2,6-Bis(5-isopropyl-5-methylimidazolidin-4-on-2-yl)pyridines Ia–c

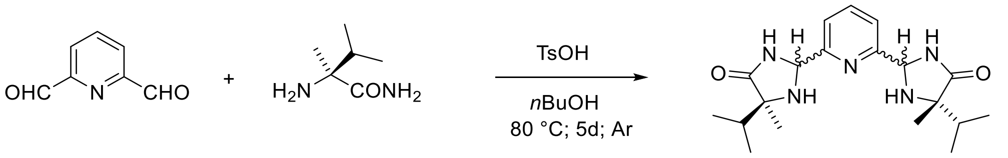

A solution of pyridine-2,6-dicarbaldehyde (271.2 mg, 2 mmol), (*S*)-2-amino-2,3-dimethylbutanamide (780 mg, 6 mmol) and TsOH (30 mg) in *n*-BuOH (6 mL) was heated at 80 °C under argon for 5 d. TEA (50 µL) was added to the cooled reaction mixture, and the solvent was evaporated under reduced pressure. The residue was at least twice chromatographed (SiO_2; acetone/AcOEt /MeOH (13/7/1; v/v/v)) to afford individual diastereomers of ligand **I**.

(2*R*,2´*R*,5*S*,5´*S*)-2,6-Bis(5-isopropyl-5-methylimidazolidin-4-on-2-yl)pyridine (Ia)

Yield: 150 mg (20%), white crystalline solid; R_f 0.51; mp 235–237 °C; $[\alpha]_D^{20}$ +32.6 (*c* 0.212; DCM/MeOH (9/1; v/v); ^{1}H NMR (500 MHz, CD_3OD): δ 7.91 (t, 1H, *J* = 7.8 Hz), 7.52 (d, 2H, *J* = 7.8 Hz), 5.54 (s, 2H), 1.93 (sp, 2H, *J* = 6.5 Hz), 1.33 (s, 6H), 1.03 (m, 12H); ^{13}C NMR (125 MHz, CD_3OD): δ 182.3, 160.6, 139.8, 123.1, 72.6, 66.4, 36.2, 24.1, 18.1, 16.7; FT-IR (ATR, cm^{-1}): 3171, 2962, 2922, 2871, 1709, 1681, 1453, 1333, 1080, 1058, 804, 627; HR-MALDI-MS (DHB): Calcd for $C_{19}H_{29}N_5O_2$ *m/z* 360.23940 [M+H]$^+$; found 360.23984.

(2*R*,2´*S*,5*S*,5´*S*)-2,6-Bis(5-isopropyl-5-methylimidazolidin-4-on-2-yl)pyridine (Ib)

Yield: 311 mg (41%), white crystalline solid; R_f 0.43; mp 208–210 °C; $[\alpha]_D^{20}$ –13.0 (*c* 0.432; DCM/MeOH (9/1; v/v); ^{1}H NMR (500 MHz, CD_3OD): δ 7.92 (t, 1H, *J* = 7.8 Hz), 7.61 (d, 1H, *J* = 7.8 Hz), 7.53 (d, 1H, *J* = 7.8 Hz), 5.64 (s, 1H), 5.55 (s, 1H), 1.94 (m, 2H), 1.36 (s, 3H), 1.34 (s, 3H), 1.03 (m, 6H); 1.00 (d, 3H, *J* = 6.8 Hz), 0.96 (d, 3H, *J* = 6.8 Hz); ^{13}C NMR (125 MHz, CD_3OD): δ 182.2, 182.0, 160.1, 160.0, 139.7, 123.2, 123.1, 72.6, 70.8, 66.5, 66.4, 36.2, 34.7, 24.0, 21.9, 18.5, 18.1, 16.7; FT-IR (ATR, cm^{-1}): 3220, 2965, 2873, 1690, 1447, 1320, 1108, 758, 666. HR-MALDI-MS (DHB): Calcd for $C_{19}H_{29}N_5O_2$ *m/z* 360.23940 $[M+H]^+$; found 360.23992.

(2*S*,2´*S*,5*S*,5´*S*)-2,6-Bis(5-isopropyl-5-methylimidazolidin-4-on-2-yl)pyridine (1c)

Yield: 115 mg (15%), white crystalline solid; R_f 0.36; mp 172–174 °C; $[\alpha]_D^{20}$ –70.0 (*c* 0.216; DCM/MeOH (9/1; v/v); ^{1}H NMR (500 MHz, CD_3OD): δ 7.95 (t, 1H, *J* = 7.8 Hz), 7.62 (d, 2H, *J* = 7.8 Hz), 5.68 (s, 2H), 1.96 (sp, 2H, *J* = 6.8 Hz), 1.38 (s, 6H), 1.01 (d, 6H, *J* = 6.8 Hz), 0.95 (d, 6H, *J* = 6.8 Hz); ^{13}C NMR (125 MHz, CD_3OD): δ 181.5, 159.6, 139.7, 123.2, 70.7, 66.7, 34.6, 21.5, 18.4, 16.7; FT-IR (ATR, cm^{-1}): 3209, 2962, 2872, 2854, 1688, 1446, 1320, 1077, 756; HR-MALDI-MS (DHB): Calcd for $C_{19}H_{29}N_5O_2$ *m/z* 360.23940 $[M+H]^+$; found 360.23976.

2,6-Bis(5-isopropyl-2,5-dimethylimidazolidin-4-on-2-yl)pyridines IIa–c

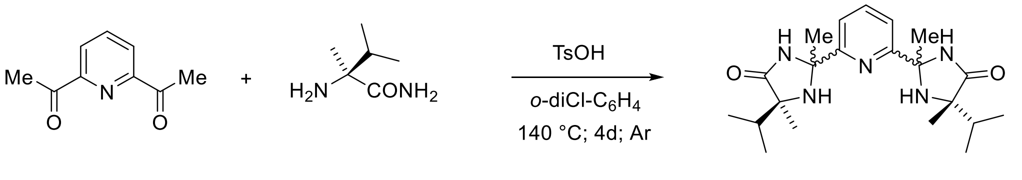

IIa (*trans-trans*)
IIb (*cis-trans*)
IIc (*cis-cis*)

A solution of 2,6-diacetylpyridine (332.8 mg, 2 mmol), (*S*)-2-amino-2,3-dimethylbutanamide (780 mg, 6 mmol) and TsOH (30 mg) in *ortho*-dichlorobenzene (6 mL) was heated at 140 °C under argon for 4 d. TEA (50 µL) was added to the cooled reaction mixture, and the solvent was evaporated under reduced pressure. The residue was at least twice chromatographed (SiO_2; AcOEt/MeOH (20:1; v/v)) to afford individual diastereomers of ligand **II**.

(2*R*,2´*R*,5*S*,5´*S*)-2,6-Bis(5-isopropyl-2,5-dimethylimidazolidin-4-on-2-yl)pyridine (IIa)

Yield: 186 mg (23%), white crystalline solid; R_f 0.39; mp 188–190 °C; $[\alpha]_D^{20}$ +80.5 (*c* 0.41; DCM); ^{1}H NMR (500 MHz, $CDCl_3$): *δ* 8.73 (bs, 2H), 7.63 (t, 1H, *J* = 8.0 Hz), 7.54 (d, 2H, *J* = 8.0 Hz), 2.30 (bs, 2H), 2.05 (sp, 2H, *J* = 6.8 Hz), 1.53 (s, 6H), 1.00 (d, 12H, *J* = 6.8 Hz), 0.97 (s, 6H); ^{13}C NMR (125 MHz, $CDCl_3$): *δ* 180.3, 164.2, 137.5, 117.7, 75.1, 66.2, 33.7, 31.4, 23.7, 18.2, 16.6; FT-IR (ATR, cm^{-1}): 3199, 2964, 2874, 1693, 1574, 1343, 1236, 1101, 949, 820, 680; HR-MALDI-MS (DHB): Calcd for $C_{21}H_{33}N_5O_2$ m/z 388.27070 $[M+H]^+$; found 388.27151.

(2*R*,2´*S*,5*S*,5´*S*)-2,6-Bis(5-isopropyl-2,5-dimethylimidazolidin-4-on-2-yl)pyridine (IIb)

Yield: 288 mg (35%), white crystalline solid; R_f 0.34; mp 172–174 °C; $[\alpha]_D^{20}$ –30.6 (*c* 0.252; DCM); ^{1}H NMR (500 MHz, $CDCl_3$): *δ* 8.41 (bs, 1H), 8.22 (bs, 1H), 7.68 (t, 1H, *J* = 7.8 Hz), 7.59 (m, 2H), 2.32 (bs, 2H), 2.03 (sp, 1H, *J* = 6.8 Hz), 1.77 (sp, 1H, *J* = 6.8 Hz), 1.69 (s, 3H), 1.63 (s, 3H), 1.43 (s, 3H), 1.06 (s, 3H), 1.01 (m, 6H), 0.92 (d, 3H, *J* = 6.8 Hz), 0.64 (d, 3H, *J* = 6.8 Hz); ^{13}C NMR (125 MHz, $CDCl_3$): *δ* 180.0, 178.8, 164.1, 163.8, 137.7, 117.6, 117.5, 75.0, 74.8, 66.0, 65.7, 34.5, 34.0, 33.5, 31.5, 24.7, 24.0, 18.3, 18.2, 16.6; FT-IR (ATR, cm^{-1}): 3196, 2965, 2873, 1697, 1574, 1445, 1364, 1237, 1111, 758, 674; HR-MALDI-MS (DHB): Calcd for $C_{21}H_{33}N_5O_2$ m/z 388.27070 $[M+H]^+$; found 388.27113.

(2*S*,2´*S*,5*S*,5´*S*)-2,6-Bis(5-isopropyl-2,5-dimethylimidazolidin-4-on-2-yl)pyridine (IIc)

Yield: 96 mg (12%), white crystalline solid; R_f 0.30; mp 141–143 °C; $[\alpha]_D^{20}$ –133.5 (*c* 0.212; DCM); ^{1}H NMR (500 MHz, $CDCl_3$): *δ* 9.45 (bs, 2H), 7.65 (t, 1H, *J* = 7.8 Hz), 7.45 (d, 2H, *J* = 7.8 Hz), 2.14 (bs, 2H), 1.75 (sp, 2H, *J* = 6.8 Hz), 1.54 (s, 6H), 1.44 (s, 6H), 0.84 (d, 6H, *J* = 6.8 Hz), 0.44 (d, 6H, *J* = 6.8 Hz); ^{13}C NMR (125 MHz, $CDCl_3$): *δ* 179.3, 163.8, 137.3, 117.2, 75.5, 66.2, 34.1, 33.9, 24.5, 18.2, 16.5; FT-IR (ATR, cm^{-1}): 3214, 2964, 2872, 1673, 1575, 1370, 1260, 1097, 793; HR-MALDI-MS (DHB): Calcd for $C_{21}H_{33}N_5O_2$ m/z 388.27070 $[M+H]^+$; found 388.27104.

5-Isopropyl-5-methyl-2-(1*H*-imidazole-2-yl)imidazolidin-4-one III

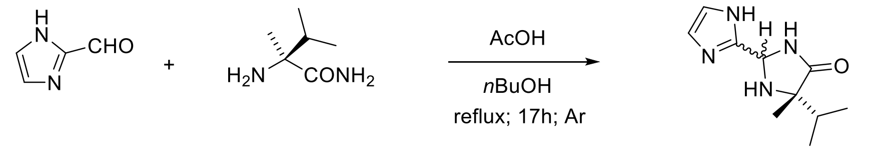

IIIa (*trans*)
IIIb (*cis*)

A solution of 1*H*-imidazole-2-carbaldehyde (380 mg, 4 mmol), (*S*)-2-amino-2,3-dimethylbutanamide (390 mg, 3 mmol) and AcOH (3 drops) in *n*-BuOH (4 mL) was heated to reflux under argon for 17 h. The reaction mixture was diluted with DCM (10 mL) and washed with a saturated solution of Na_2CO_3 (10 mL). The organic layer was dried over Na_2SO_4 and evaporated under reduced pressure. The residue was chromatographed (SiO_2; acetone) to afford individual diastereomers of ligand **III**.

(2*R*,5*S*)-5-Isopropyl-5-methyl-2-(1*H*-imidazol-2-yl)imidazolidin-4-one IIIa

Yield: 175 mg (28%), yellow crystalline solid; R_f 0.25; mp 82–84 °C; $[\alpha]_D^{20}$ –8.5 (*c* 0.52; DCM); ^1H NMR (500 MHz, $CDCl_3$): δ 8.53 (bs, 1H), 6.98 (s 2H), 5.60 (s, 1H), 1.89 (sp, 1H, *J* = 6.8 Hz), 1.20 (s, 3H), 0.95 (d, 6H, *J* = 6.8 Hz); ^{13}C NMR (125 MHz, $CDCl_3$): δ 180.6, 148.0, 122.7, 65.3, 64.8, 34.9, 23.7, 17.9, 16.5; HR-MALDI-MS (DHB): Calcd for $C_{10}H_{16}N_4O$ m/z 209.13969 $[M+H]^+$; found 209.13972.

(2*S*,5*S*)-5-Isopropyl-5-methyl-2-(1*H*-imidazol-2-yl)imidazolidin-4-one IIIb

Yield: 237 mg (38%), yellow oil; R_f 0.17; $[\alpha]_D^{20}$ –34.1 (*c* 0.50; DCM); ^1H NMR (500 MHz, $CDCl_3$): δ 7.47 (bs, 1H), 7.03 (s 2H), 5.72 (s, 1H), 1.93 (sp, 1H, *J* = 6.8 Hz), 1.33 (s, 3H), 0.95 (d, 3H, *J* = 6.8 Hz), 0.90 (d, 3H, *J* = 6.8 Hz); ^{13}C NMR (125 MHz, $CDCl_3$): δ 178.9, 147.5, 122.9, 64.6, 63.4, 33.7, 22.3, 18.3, 16.5; HR-MALDI-MS (DHB): Calcd for $C_{10}H_{16}N_4O$ m/z 209.13969 $[M+H]^+$; found 209.13968.

(*S*)-1-Cbz-Pyrrolidine-2-carbaldehyde

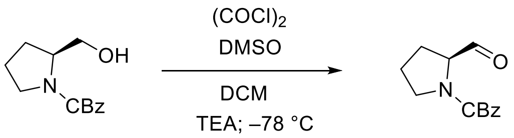

(*S*)-1-Cbz-Pyrrolidine-2-carbaldehyde was prepared according to the method described in Ref. [4]. To a solution of DMSO (3.32 mL, 50 mmol, 3 equiv) in dry DCM (35 mL) was added dropwise a solution of oxalyl chloride (3.3 mL, 37.5 mmol, 2.25 equiv) in dry DCM (15 mL) at –78 °C. The reaction mixture was stirred for 45 min and then, a solution of (*S*)-(1-Cbz-pyrrolidine-2-yl) methanol (3.05 mL, 16.7 mmol) in dry DCM (20 mL) was over 20 min. After being stirred for an additional 20 min, TEA (9.4 mL; 67 mmol, 4 equiv) was added, the reaction mixture was gradually heated at 0 °C and stirred for 2 h. After the addition of DCM (75 mL), the mixture was washed with a saturated solution of $NaHCO_3$ (75 mL) and brine (75 mL). The organic layer was dried over Na_2SO_4 and evaporated under reduced pressure to give 2.92 g (75%) of aldehyde as a yellow oil, which was introduced into the next step without further purification. ^1H NMR (500 MHz, $CDCl_3$): δ 9.54 (d, 1H, J = 8.0 Hz), 7.38–7.31 (m, 5H), 5.29–5.13 (m, 2H), 4.30–4.19 (m, 1H), 3.60–3.51 (m, 2H), 2.17–2.00 (m, 2H), 1.94–1.85 (m, 2H).

(*S*)-2-(1-CBz-pyrrolidine-2-yl)-1*H*-imidazole

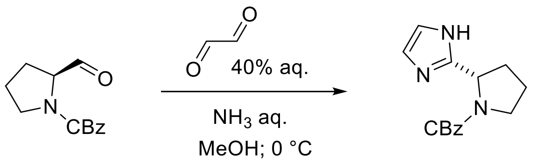

(*S*)-2-(1-CBz-pyrrolidine-2-yl)-1*H*-imidazole was prepared according to the method described in Ref. [2]. To a solution of (*S*)-1-Cbz-pyrrolidine-2-carbaldehyde (2.62 g, 12.2 mmol) in MeOH (20 mL) was added 40% aqueous solution of glyoxal (4.8 mL, 42 mmol, 3.5 equiv) and 26% aqueous solution of ammonia (5.6 mL, 70 mmol, 5.75 equiv) at 0 °C. The reaction mixture was stirred at room temperature for 24 hours. The solvents were evaporated under reduced pressure, and the residue was mixed with AcOEt (50 mL). The precipitate was removed by

filtration, and the organic phase was washed with a saturated solution of $NaHCO_3$ (2 × 25 mL). The organic layer was dried over Na_2SO_4 and evaporated under reduced pressure. The crude product was purified by column chromatography (R_f 0.41; SiO_2; acetone) to give the imidazole derivative as a white crystalline solid (1.36 g; 48%); mp 118–119 °C; $[\alpha]_D^{20}$ = –58.7 (*c* 1.006; MeOH); ^{1}H NMR (500 MHz, $CDCl_3$): δ 10.37 (bs, 1H), 7.38–7.31 (m, 5H), 6.96 (s, 2H), 5.18 (d, 1H, *J* = 10.0 Hz), 5.13 (d, 1H, *J* = 10.0 Hz), 4.99 (m, 1H), 3.48 (m, 2H), 2.94 (m, 1H), 2.19–2.13 (m, 2H), 1.98 (m, 1H); ^{13}C NMR (125 MHz, $CDCl_3$): δ 156.9, 148.4, 148.1, 136.6, 128.8, 128.4, 128.0, 67.5, 54.7, 47.3, 28.3, 25.1; FT-IR (ATR, cm^{-1}): 1697, 1456, 1408, 1348, 1099, 955, 773, 741, 731, 696; HR-MALDI-MS (DHB): Calcd for $C_{15}H_{17}N_3O_2$ *m/z* 272.13936 $[M+H]^+$; found 272.13971.

(*S*)-2-(Pyrrolidine-2-yl)-1*H*-imidazole (IV)

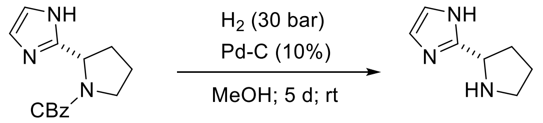

To a solution of (*S*)-2-(1-CBz-pyrrolidine-2-yl)-1*H*-imidazole (813 mg, 3.21 mmol) in EtOH (20 mL) was carefully added 10% Pd/C (318 mg, 0.3 mmol). The mixture was stirred under an atmosphere of hydrogen (40 bar) at room temperature for 5 d. After this period, the stream of argon was introduced to the mixture (ca. 5 min), the catalyst was removed by filtration and washed with MeOH (50 mL). The solvents were evaporated under reduced pressure to give compound **4** (0.42 g; 97%) as a yellow oil; $[\alpha]_D^{20}$ = –41.2 (*c* 1.00; MeOH); ^{1}H NMR (500 MHz, $CDCl_3$): δ 7.15 (bs, 2H), 6.94 (s, 2H), 4.30 (d, 1H, *J* = 7.0 Hz), 3.03–2.96 (m, 1H), 2.94–2.91 (m, 1H), 2.21–2.14 (m, 1H), 2.11–2.04 (m, 1H), 1.88–1.83 (m, 2H); ^{13}C NMR (125 MHz, $CDCl_3$): δ 149.8, 122.1, 56.2, 46.5, 31.9, 25.6; FT-IR (ATR, cm^{-1}): 2972, 1697, 1406, 1254, 1099, 741, 696; HR-MALDI-MS (DHB): Calcd for $C_7H_{117}N_3$ *m/z* 138.10257 $[M+H]^+$; found 138.10253.

General procedure for asymmetric Henry reaction

A mixture of ligand **I–IV** (27.5 μmol), $Cu(OAc)_2$ (4.5 mg, 25 μmol) and nitromethane (0.5 mL, 9 mmol) in absolute ethanol (1 mL) was stirred for 1 h at room temperature. Then the mixture was cooled at 10 °C, aldehyde (0.5 mmol) was added, and a formed solution was stirred for the 2 d. The solvents were removed under reduced pressure, and the crude product was analysed by ^{1}H NMR for determination of conversion. The nitroalcohol was purified by column or flash chromatography (AcOEt/hexane; 1:3 (v/v)**.** The enantiomeric excess was determined by HPLC. The characterisation data for corresponding nitroalcohols were in accordance with data published previously [1,6].

General procedure for asymmetric aldol reaction

To a solution of catalyst **IV** (13.6, 0.1 mmol) and TFA (0.1 mmol or 0.2 mmol) in an appropriate solvent (2 mL) was added cyclohexanone or acetone (5 mmol). The reaction mixture was cooled to the corresponding temperature, and the aldehyde (0.5 mmol) was added. The mixture was kept at the used temperature for the chosen reaction time. The catalyst was removed by flash chromatography (SiO_2; PE/AcOEt 1:1 (v/v)) and the solvent and excess ketone were evaporated under reduced pressure. The crude product was analysed by ^{1}H NMR for determination of diastereomeric ratio. The aldol was purified by column or flash chromatography (AcOEt/hexane; 1:1 (v/v)**.** The enantiomeric excess in the major diastereomer was determined by HPLC. The characterisation data for corresponding aldols were in accordance with data published previously [7,8].

3. NMR spectra

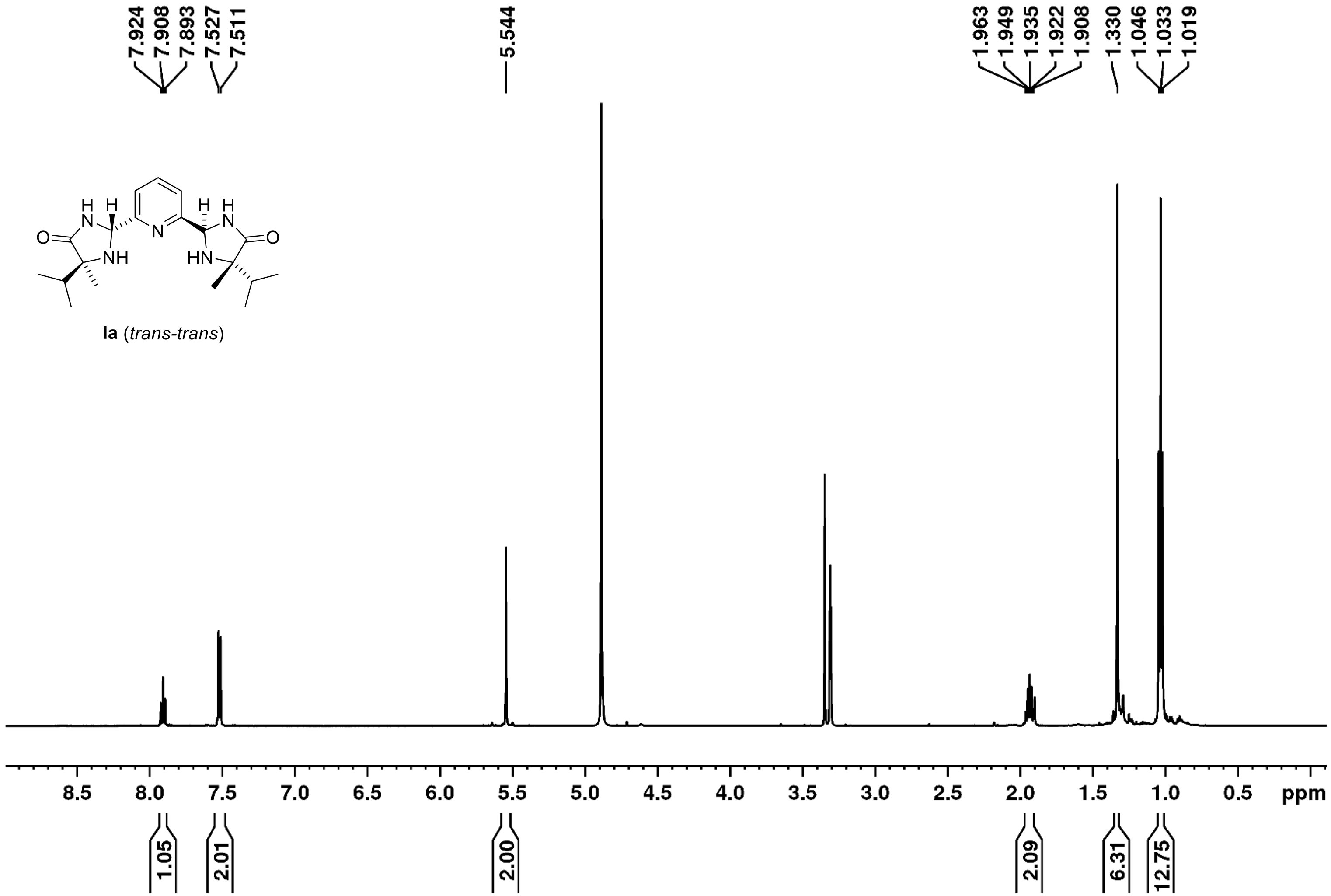
7.924
7.908
7.893
7.527
7.511
5.544
1.963
1.949
1.935
1.922
1.908
1.330
1.046
1.033
1.019
Ia (*trans-trans*)
8.5 8.0 7.5 7.0 6.5 6.0 5.5 5.0 4.5 4.0 3.5 3.0 2.5 2.0 1.5 1.0 0.5 ppm
1.05
2.01
2.00
2.09
6.31
12.75

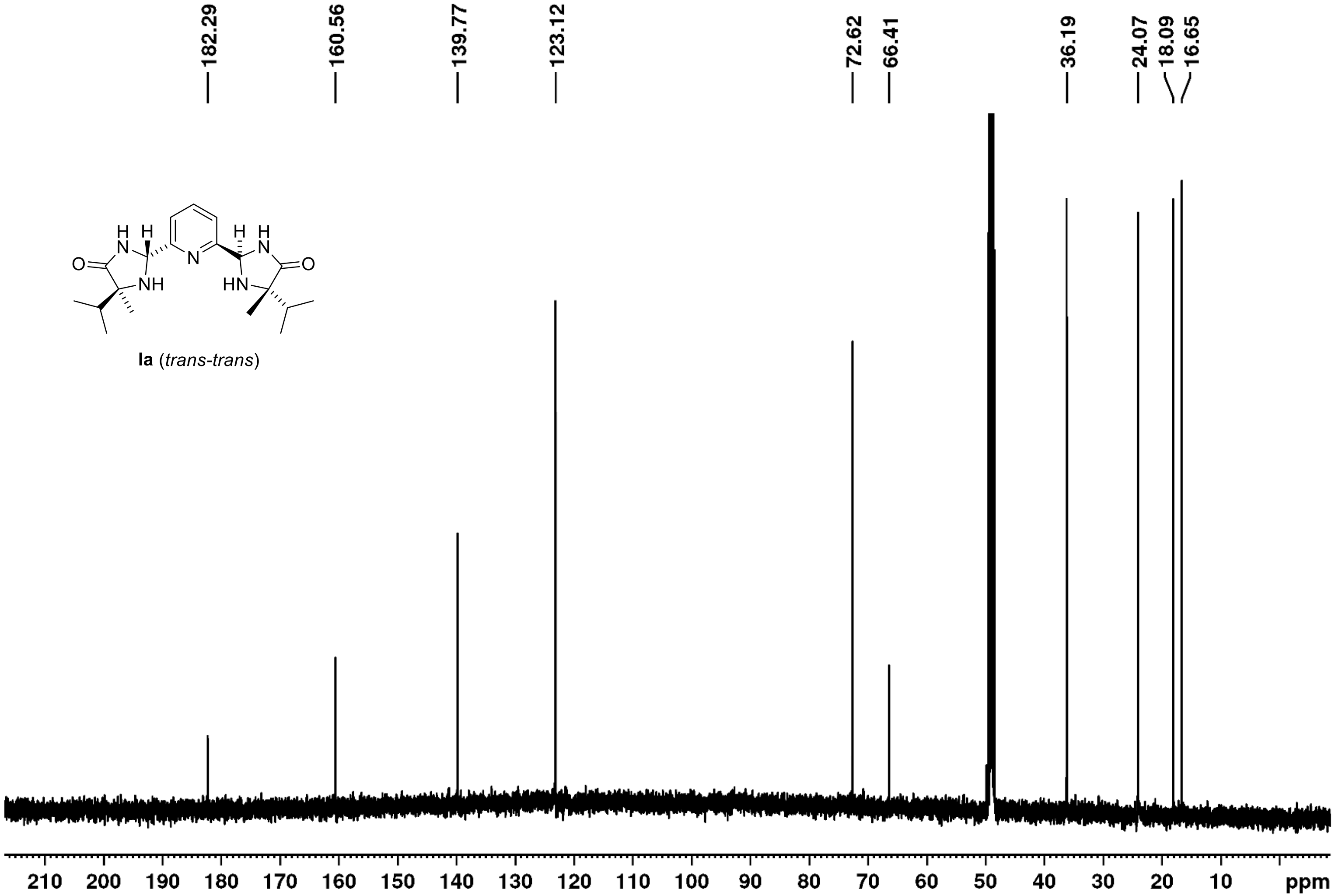
182.29
160.56
139.77
123.12
72.62
66.41
36.19
24.07
18.09
16.65
Ia (trans-trans)
210 200 190 180 170 160 150 140 130 120 110 100 90 80 70 60 50 40 30 20 10 ppm

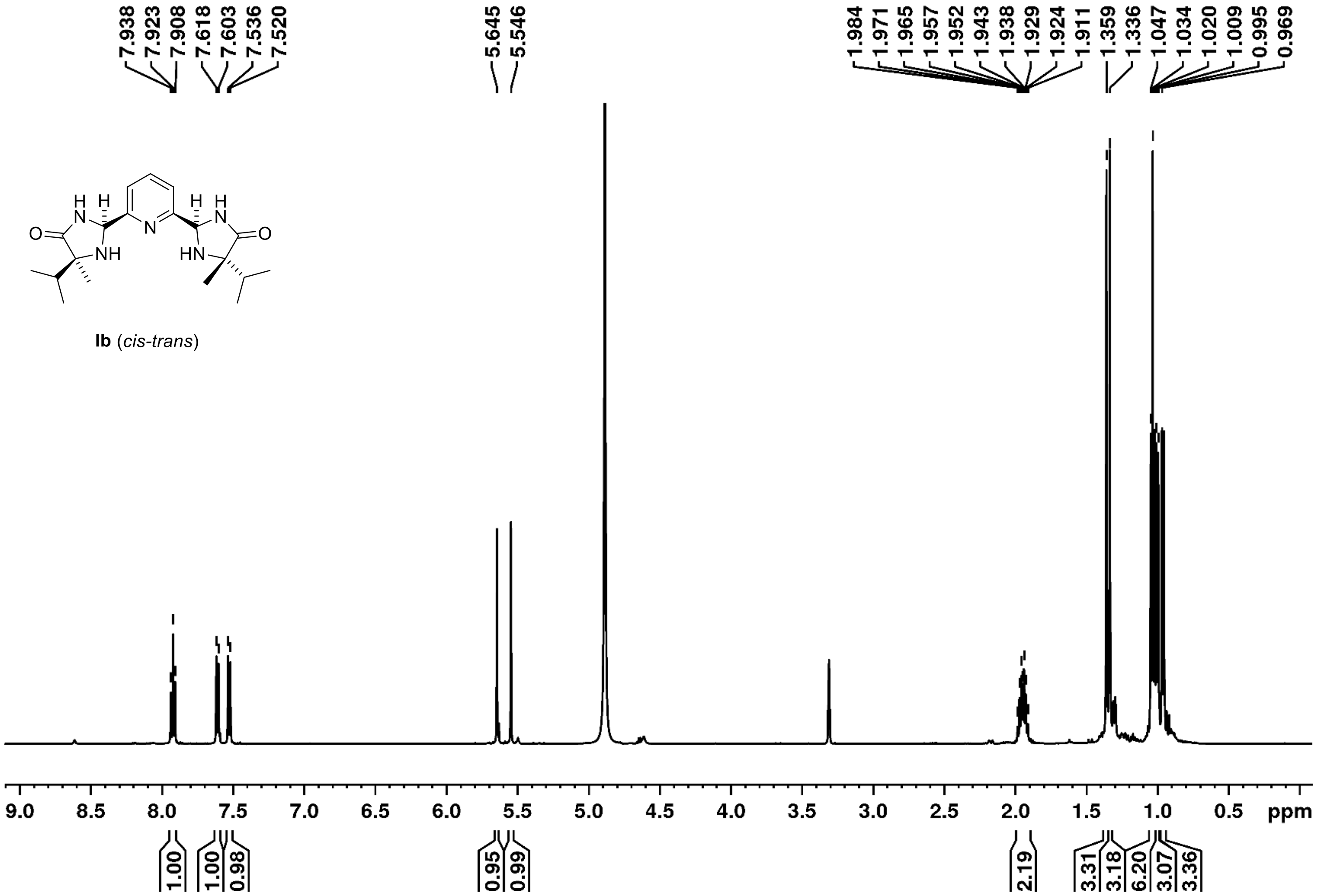
7.938
7.923
7.908
7.618
7.603
7.536
7.520
5.645
5.546
1.984
1.971
1.965
1.957
1.952
1.943
1.938
1.929
1.924
1.911
1.359
1.336
1.047
1.034
1.020
1.009
0.995
0.969
H
H
H
H
N
N
O
NH
HN
O
N
9.0
8.5
8.0
7.5
7.0
6.5
6.0
5.5
5.0
4.5
4.0
3.5
3.0
2.5
2.0
1.5
1.0
0.5
ppm
1.00
1.00
0.98
0.95
0.99
2.19
3.31
3.18
6.20
3.07
3.36
Ib (*cis-trans*)

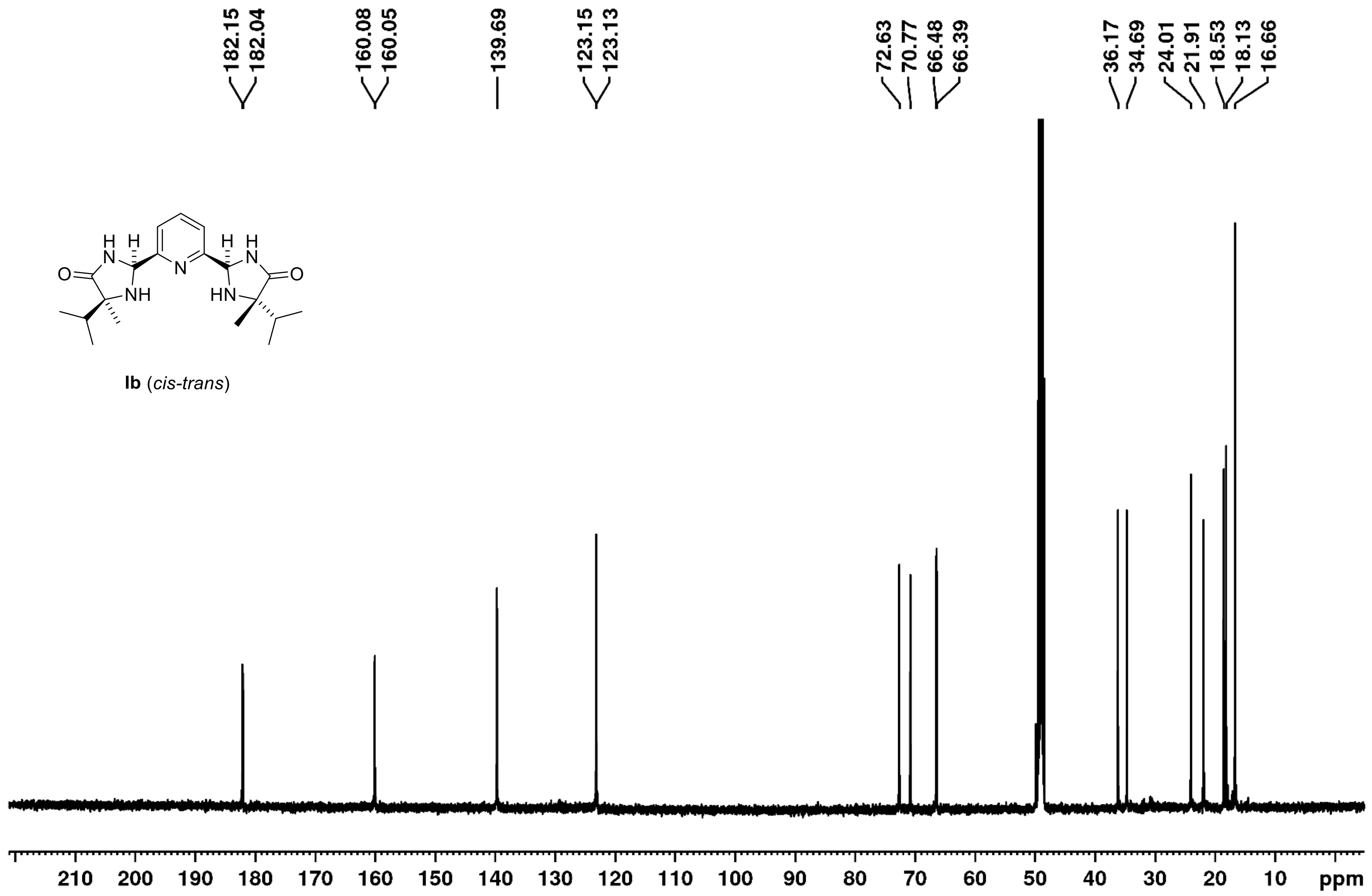
182.15
182.04
160.08
160.05
139.69
123.15
123.13
72.63
70.77
66.48
66.39
36.17
34.69
24.01
21.91
18.53
18.13
16.66
210
200
190
180
170
160
150
140
130
120
110
100
90
80
70
60
50
40
30
20
10
ppm
O
N
H
NH
HN
O

Ib (*cis-trans*)

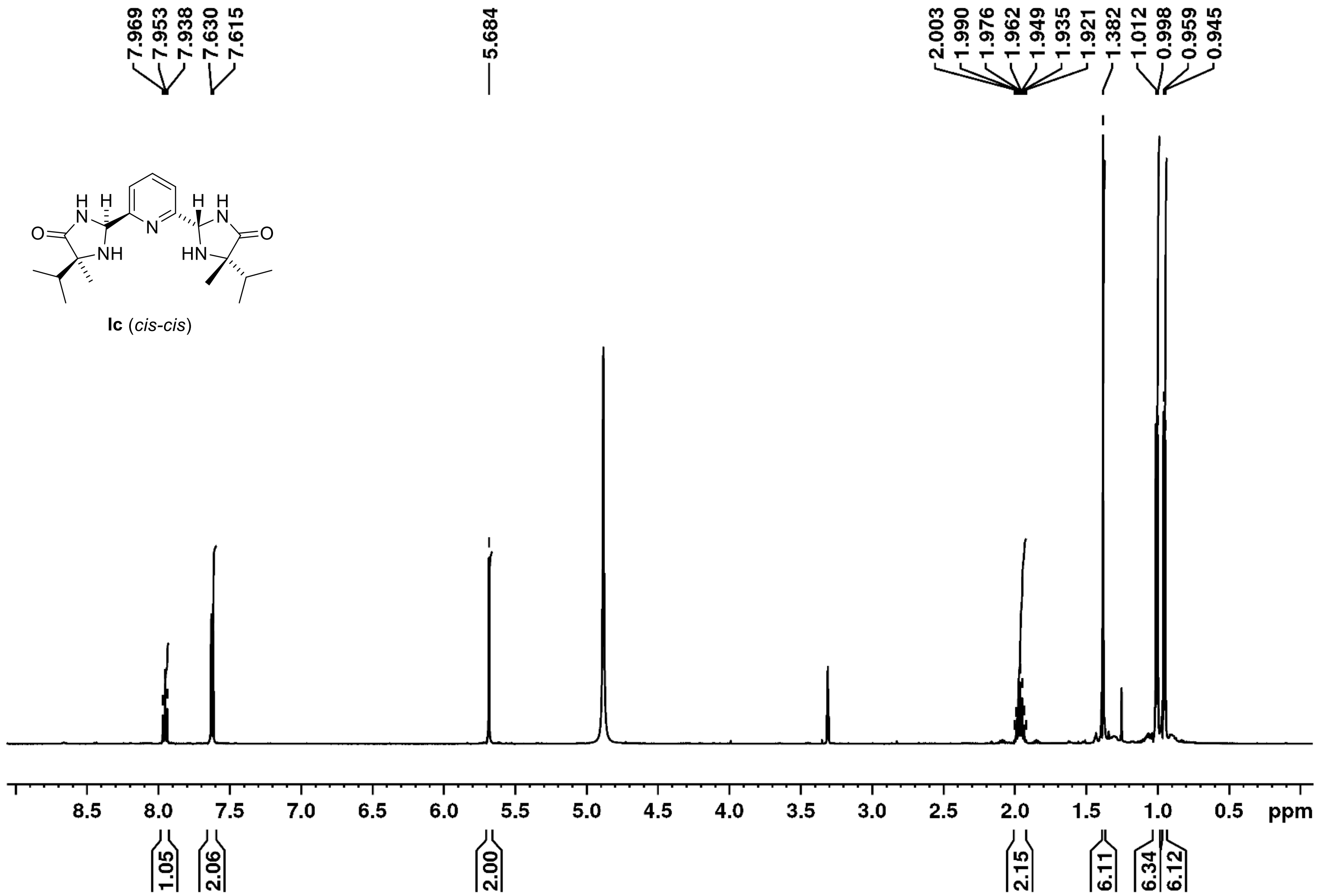
7.969
7.953
7.938
7.630
7.615
5.684
2.003
1.990
1.976
1.962
1.949
1.935
1.921
1.382
1.012
0.998
0.959
0.945
H
H
H
H
N
O
NH
HN
O
Ic (cis-cis)
8.5
8.0
7.5
7.0
6.5
6.0
5.5
5.0
4.5
4.0
3.5
3.0
2.5
2.0
1.5
1.0
0.5
ppm
1.05
2.06
2.00
2.15
6.11
6.34
6.12

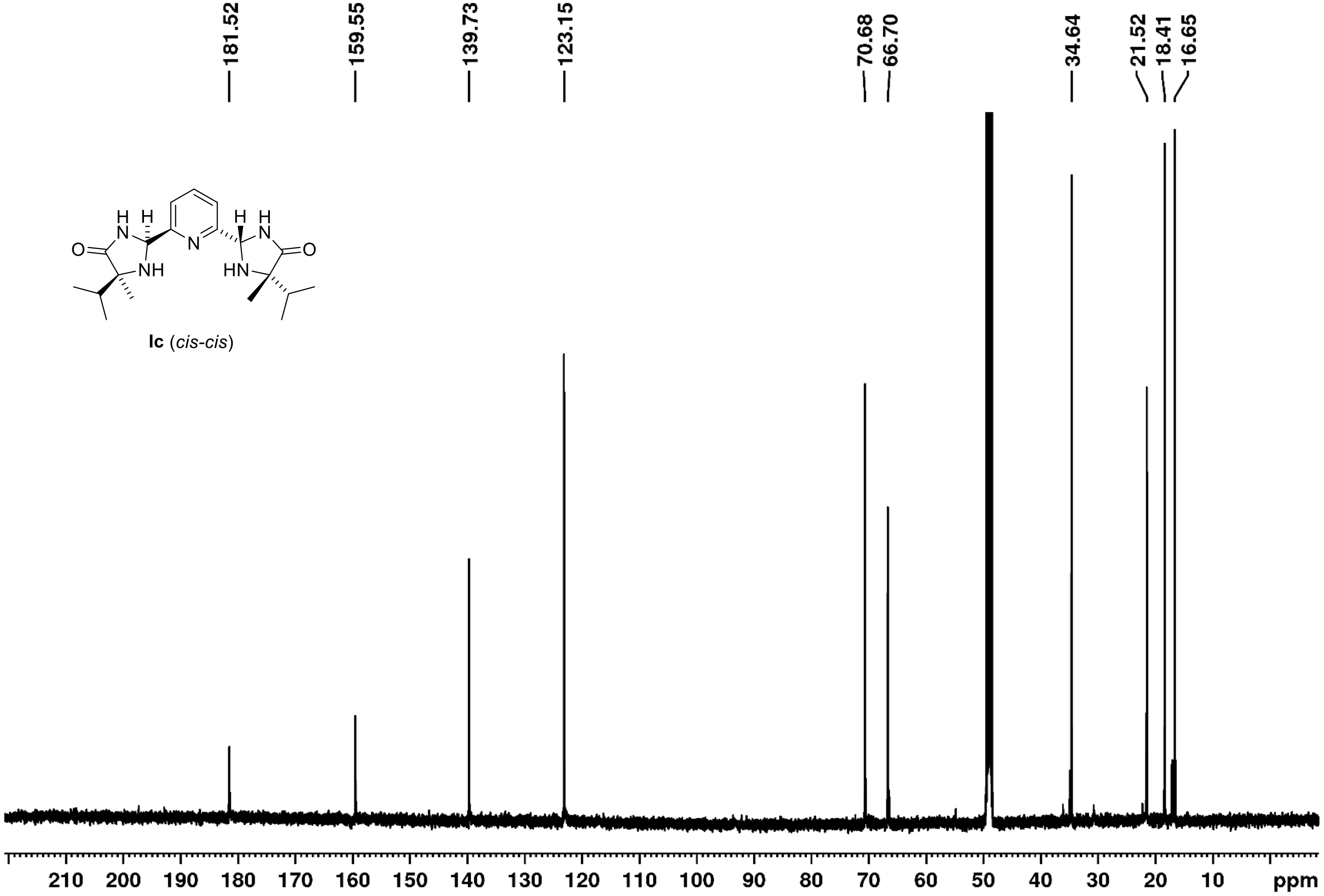
181.52
159.55
139.73
123.15
70.68
66.70
34.64
21.52
18.41
16.65
Ic (*cis*-*cis*)
210 200 190 180 170 160 150 140 130 120 110 100 90 80 70 60 50 40 30 20 10 ppm

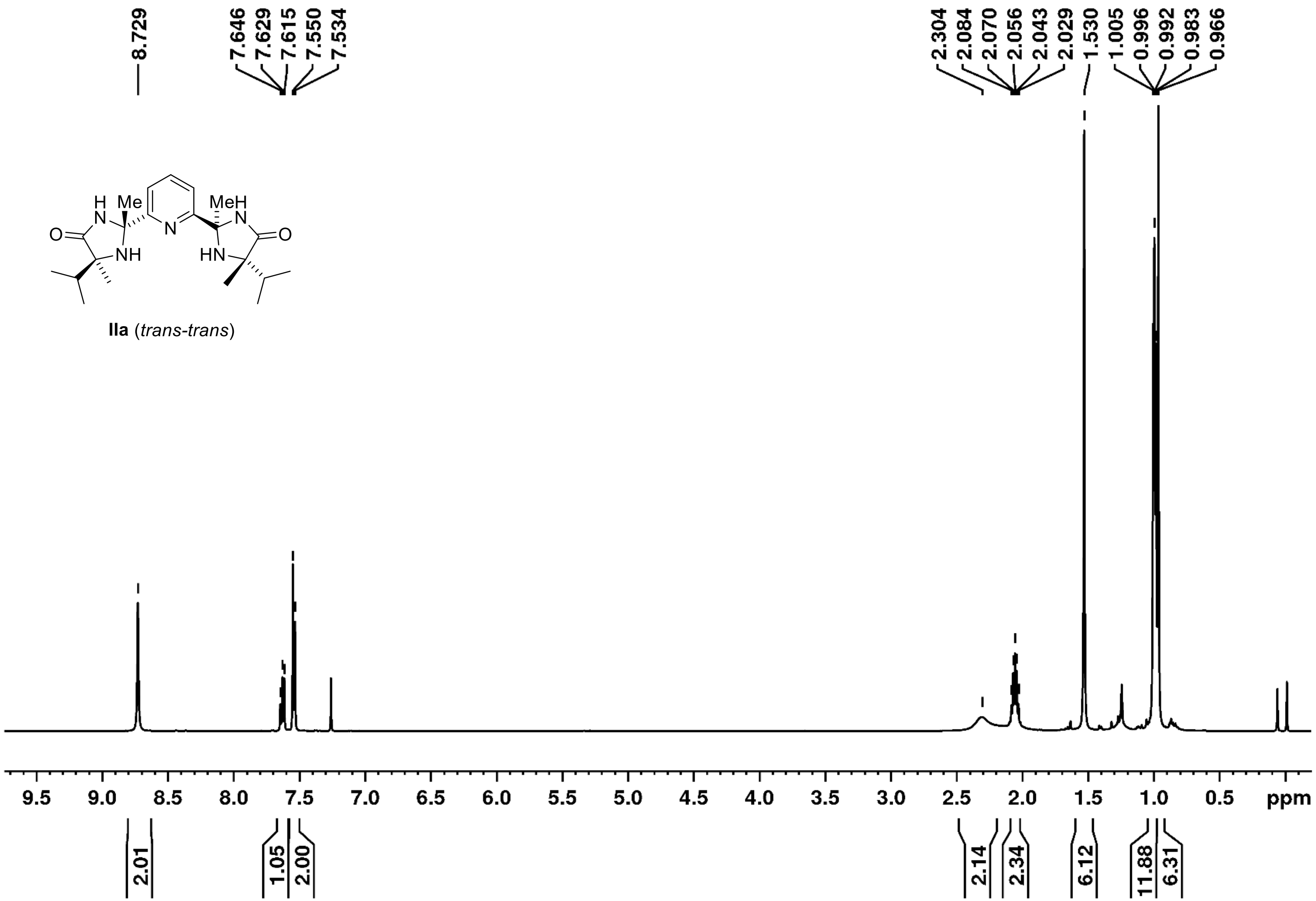
8.729
7.646
7.629
7.615
7.550
7.534
2.304
2.084
2.070
2.056
2.043
2.029
1.530
1.005
0.996
0.992
0.983
0.966
IIa (trans-trans)
9.5 9.0 8.5 8.0 7.5 7.0 6.5 6.0 5.5 5.0 4.5 4.0 3.5 3.0 2.5 2.0 1.5 1.0 0.5 ppm
2.01
1.05
2.00
2.14
2.34
6.12
11.88
6.31

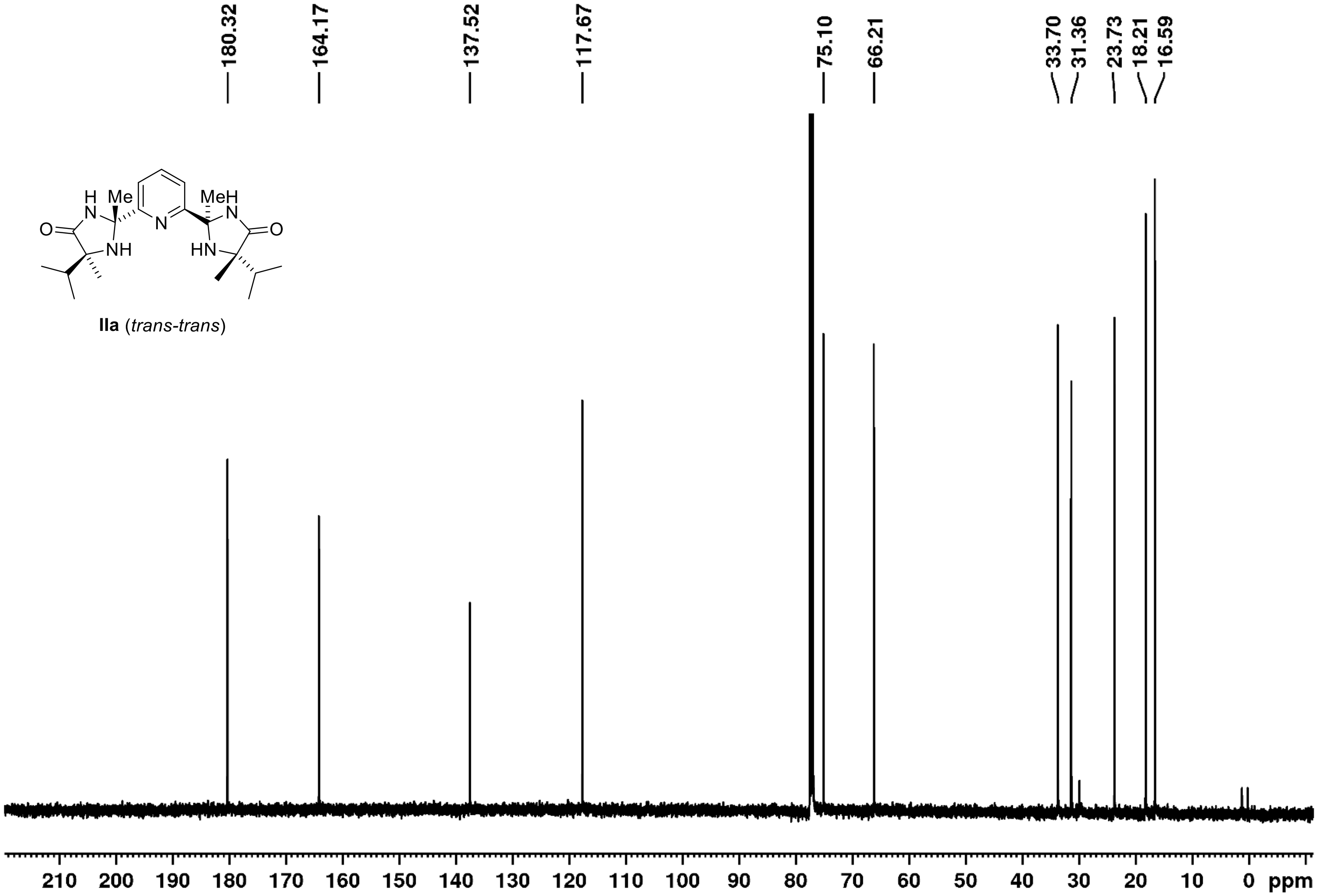

IIa (*trans-trans*)

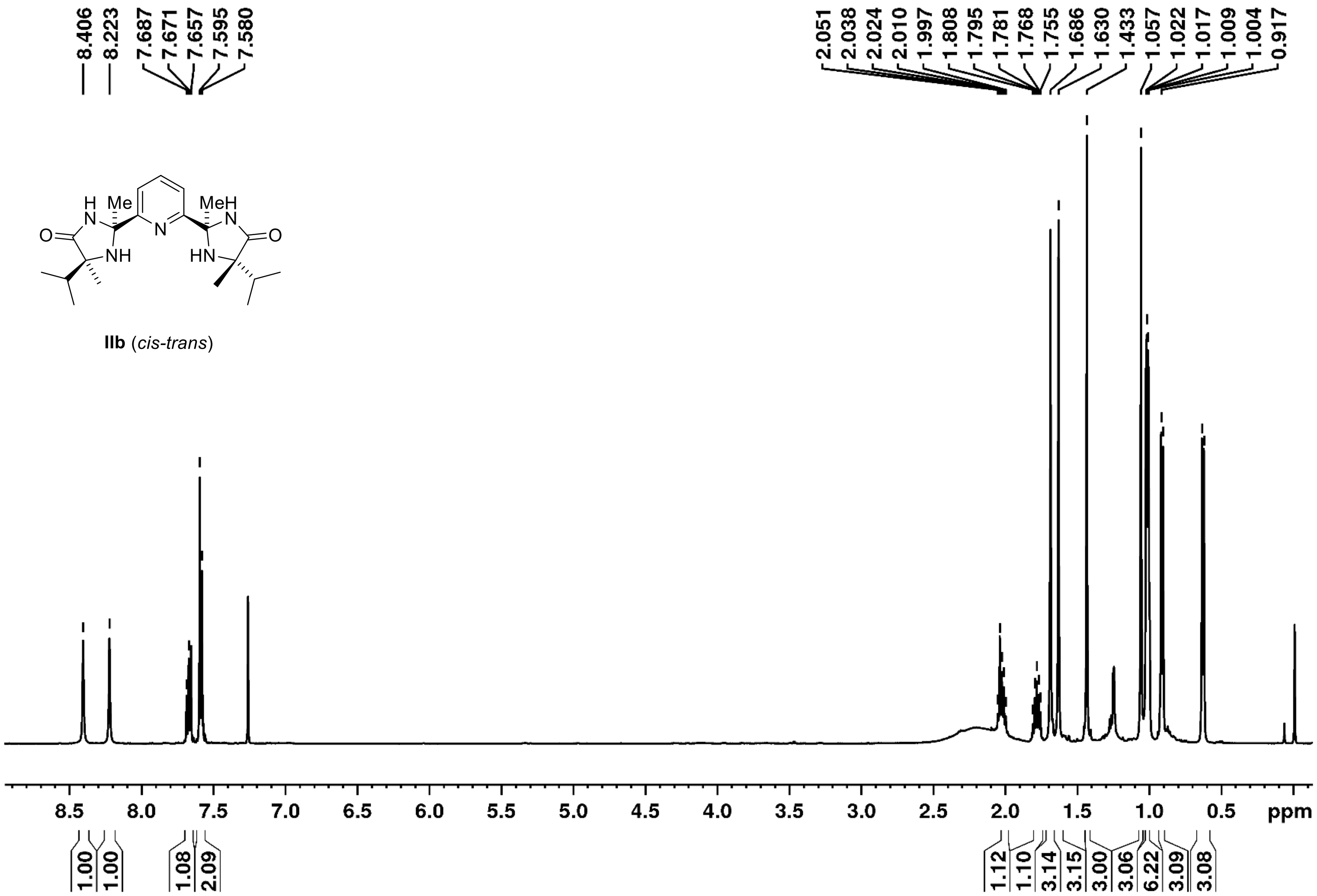
8.406
8.223
7.687
7.671
7.657
7.595
7.580
2.051
2.038
2.024
2.010
1.997
1.808
1.795
1.781
1.768
1.755
1.686
1.630
1.433
1.057
1.022
1.017
1.009
1.004
0.917
H
Me
N
O
NH
N
MeH
N
O
HN
IIb (cis-trans)
8.5
8.0
7.5
7.0
6.5
6.0
5.5
5.0
4.5
4.0
3.5
3.0
2.5
2.0
1.5
1.0
0.5
ppm
1.00
1.00
1.08
2.09
1.12
1.10
3.14
3.15
3.00
3.06
6.22
3.09
3.08

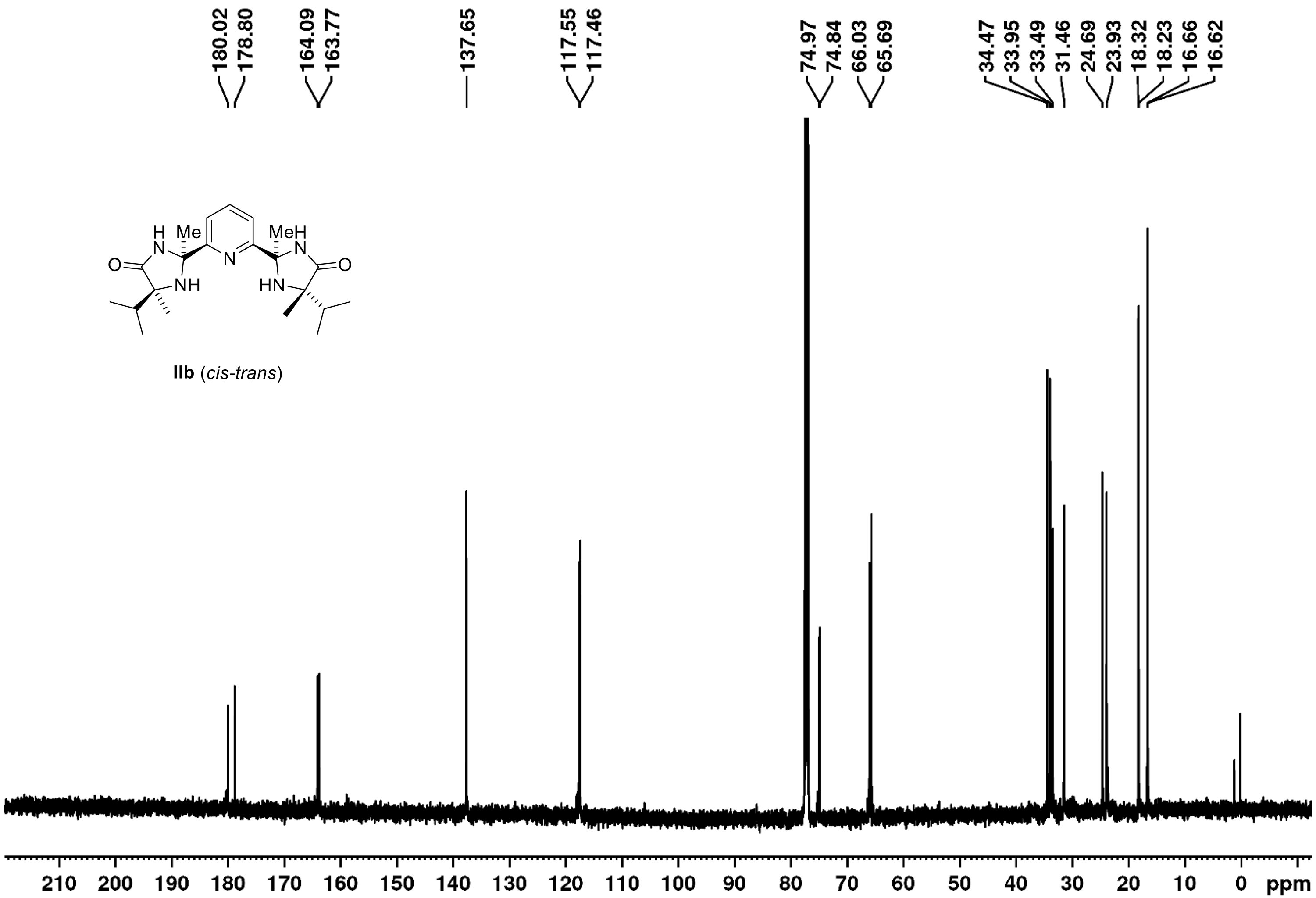

IIb (*cis-trans*)

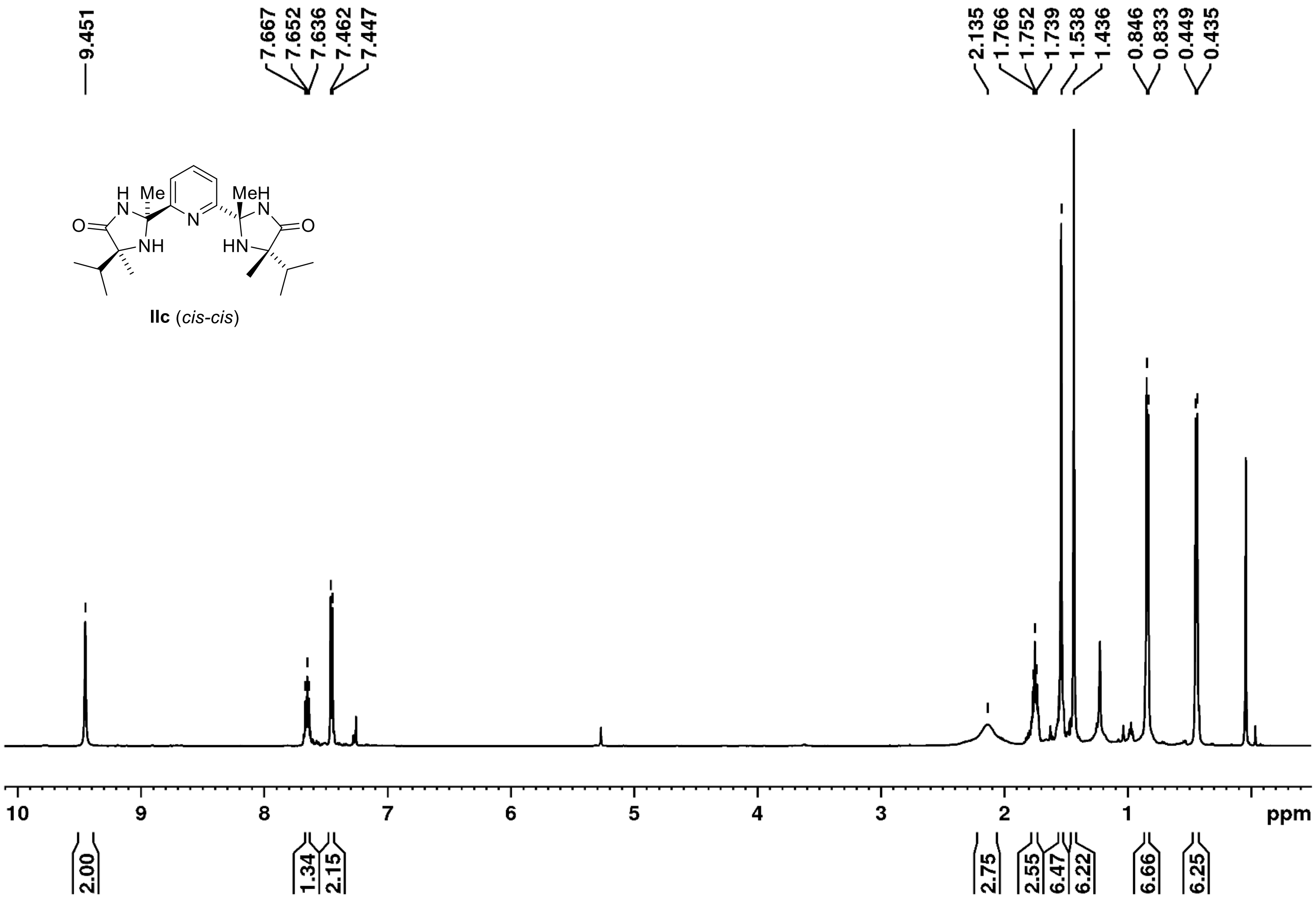

9.451
7.667
7.652
7.636
7.462
7.447
2.135
1.766
1.752
1.739
1.538
1.436
0.846
0.833
0.449
0.435
H
Me
MeH
N
O
NH
HN
IIc (cis-cis)
10
9
8
7
6
5
4
3
2
1
ppm
2.00
1.34
2.15
2.75
2.55
6.47
6.22
6.66
6.25

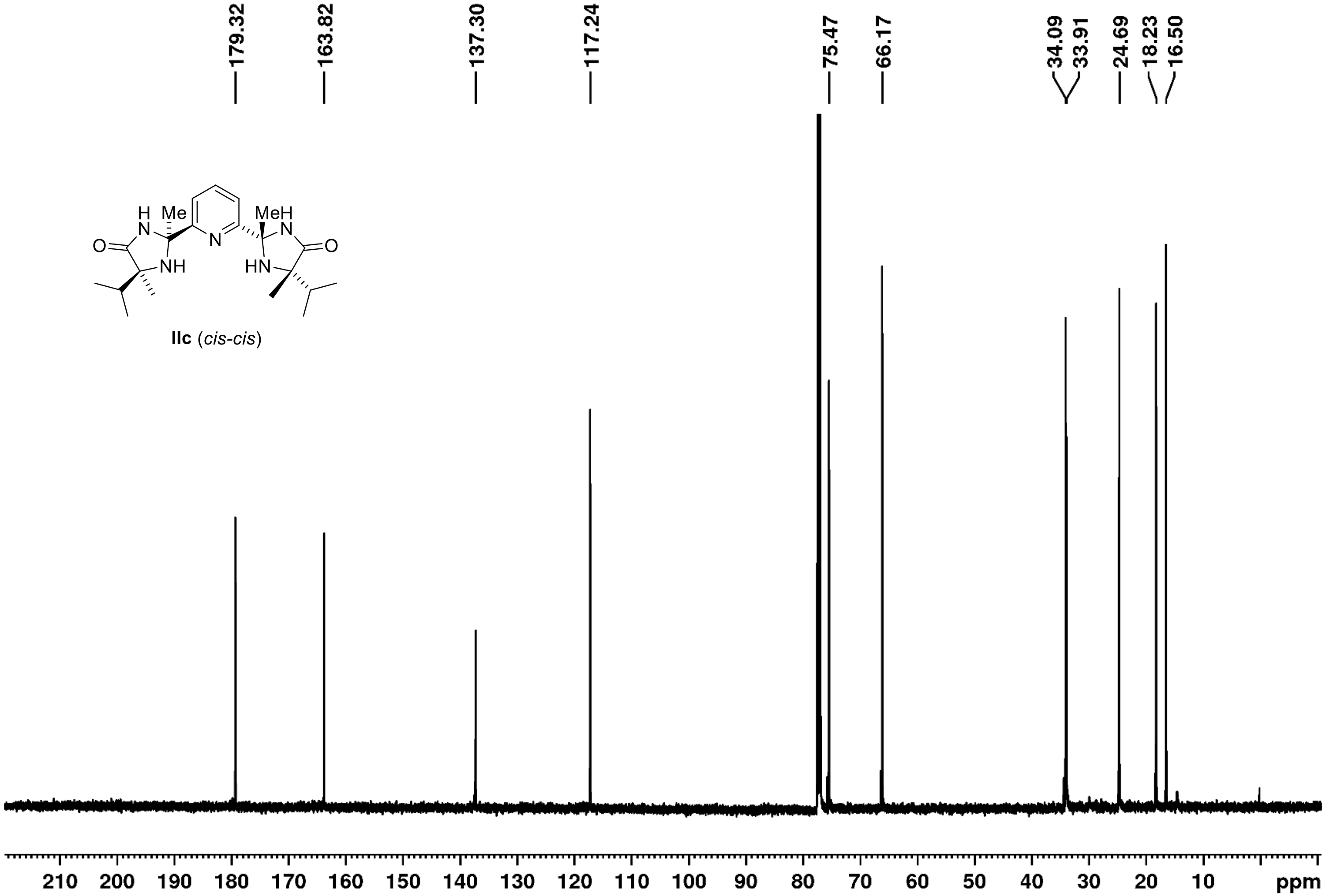
179.32
163.82
137.30
117.24
75.47
66.17
34.09
33.91
24.69
18.23
16.50
H
N
Me
MeH
N
O
N
O
NH
HN
IIc (cis-cis)
210 200 190 180 170 160 150 140 130 120 110 100 90 80 70 60 50 40 30 20 10 ppm

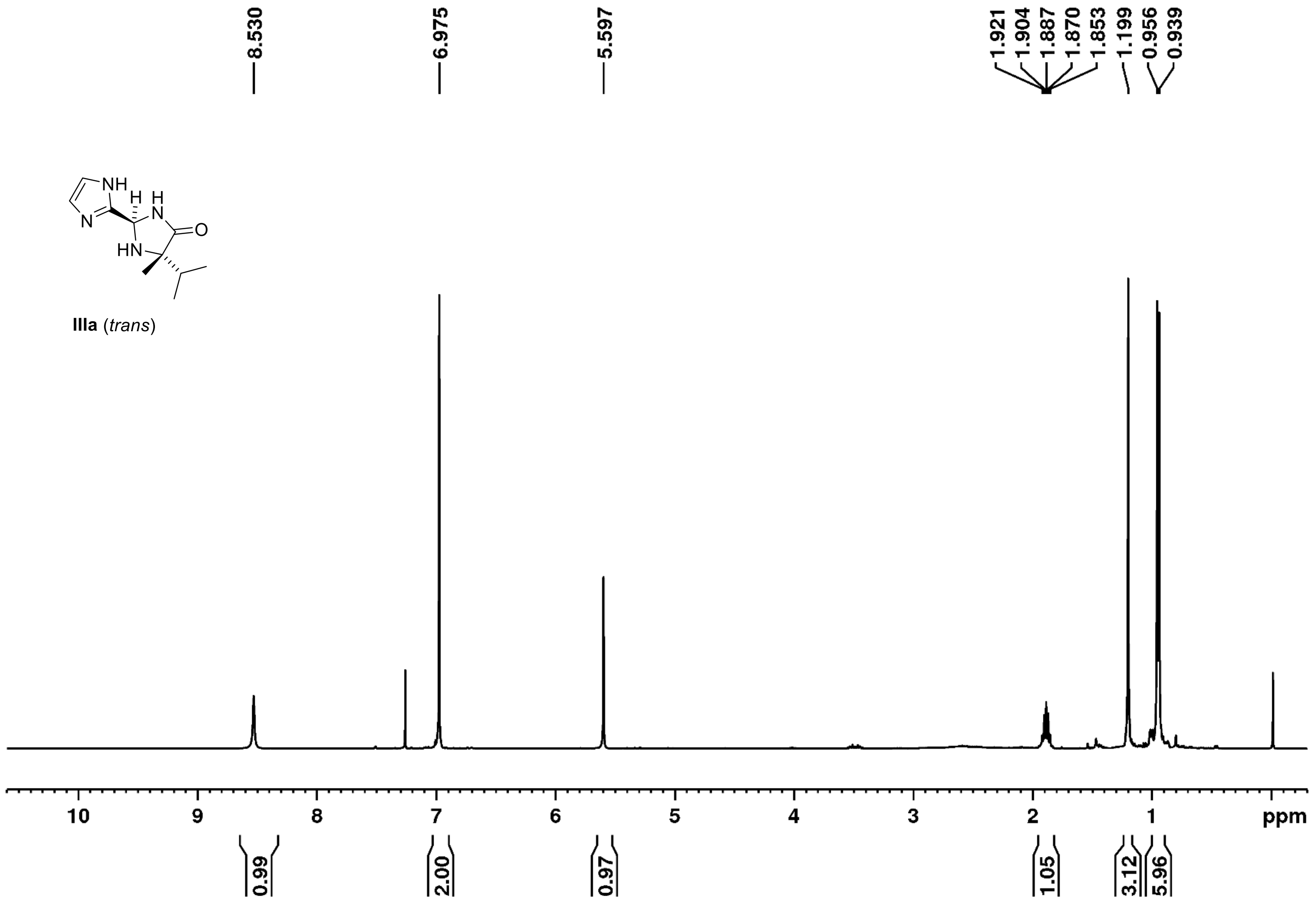

8.530
6.975
5.597
1.921
1.904
1.887
1.870
1.853
1.199
0.956
0.939
IIIa (*trans*)
10
9
8
7
6
5
4
3
2
1
ppm
0.99
2.00
0.97
1.05
3.12
5.96

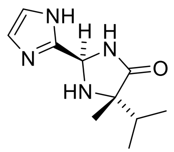

IIIa (*trans*)

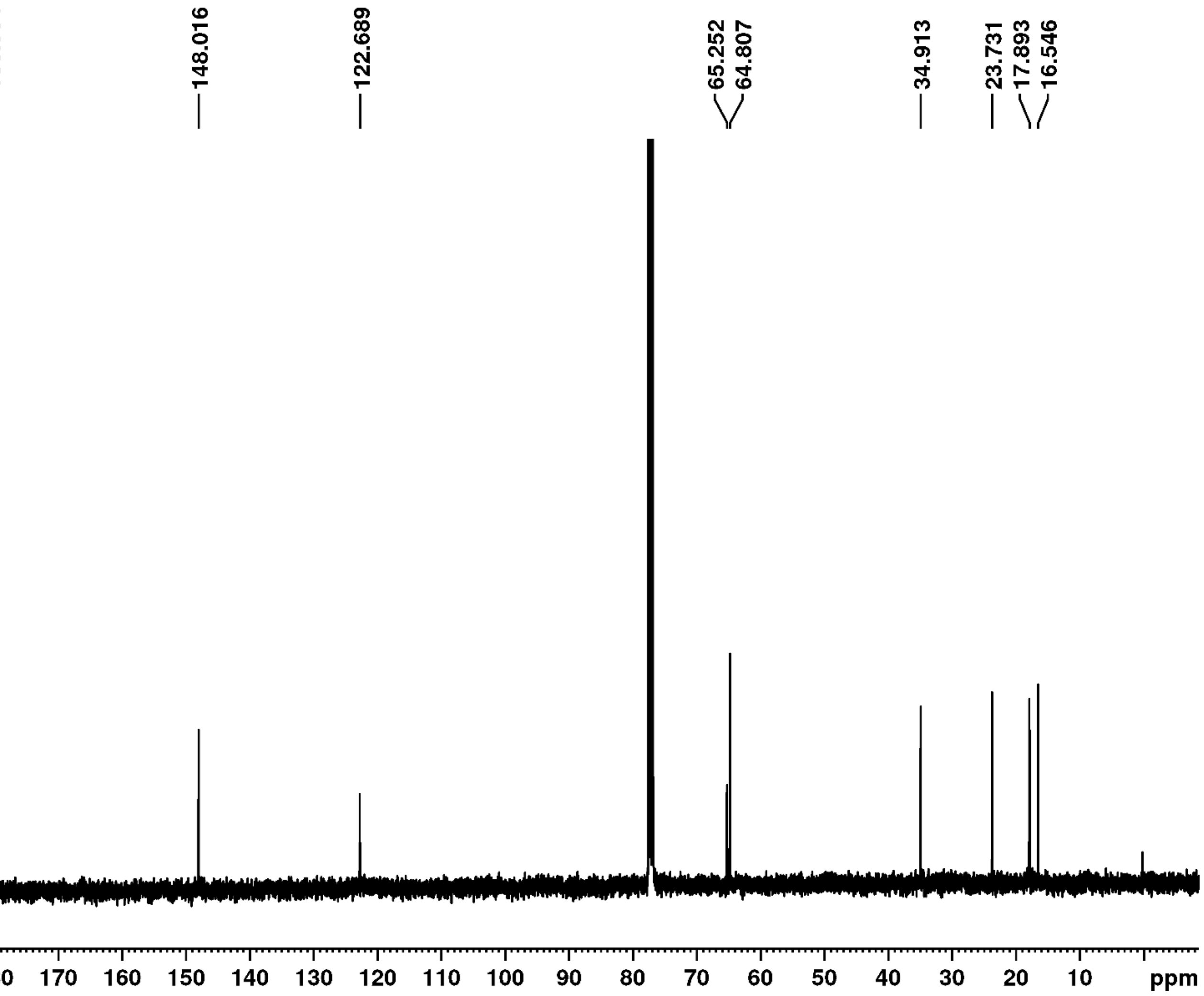
180.564
148.016
122.689
65.252
64.807
34.913
23.731
17.893
16.546
210 200 190 180 170 160 150 140 130 120 110 100 90 80 70 60 50 40 30 20 10 ppm

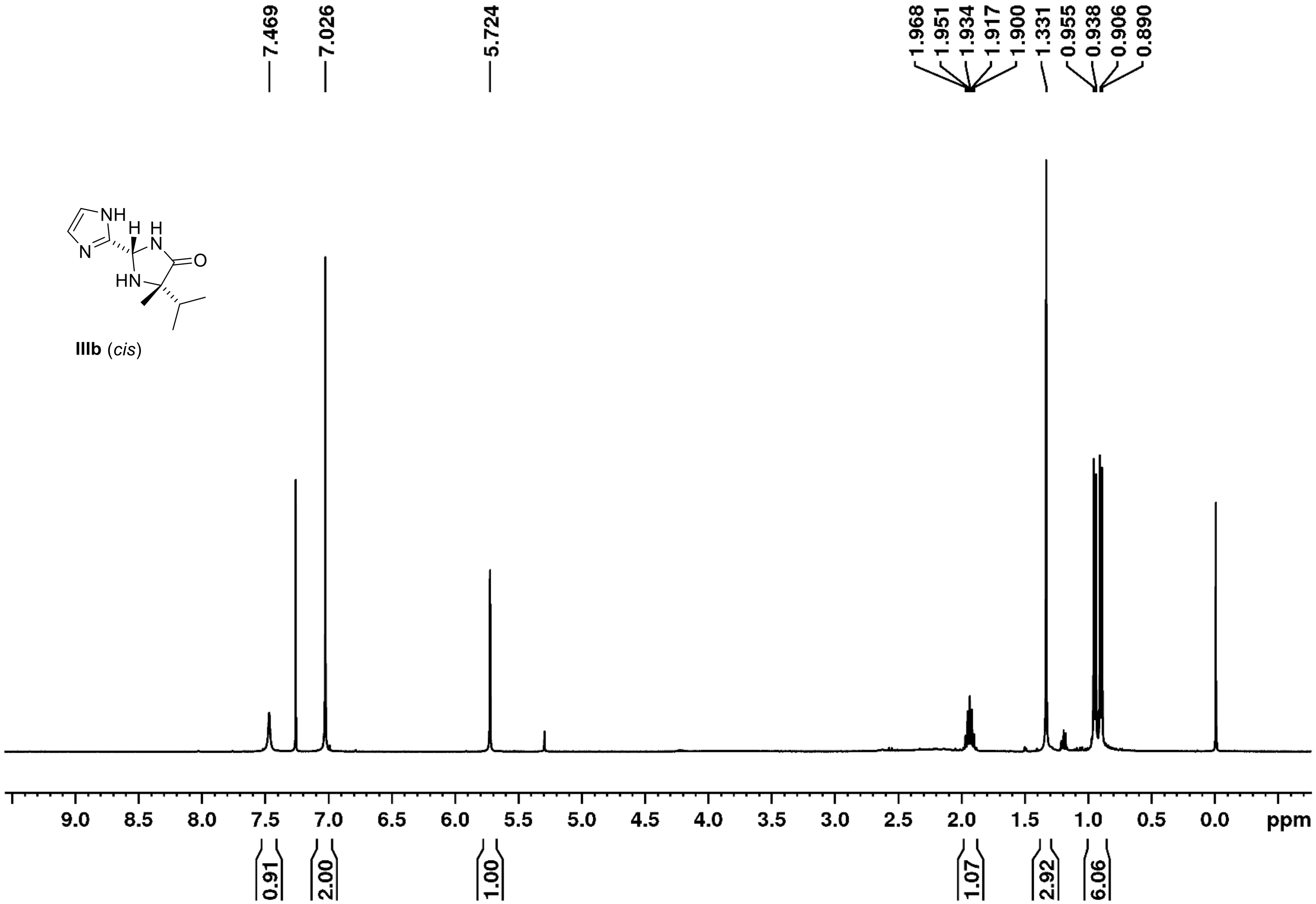
7.469
7.026
5.724
1.968
1.951
1.934
1.917
1.900
1.331
0.955
0.938
0.906
0.890
NH
H
H
N
N
O
HN
9.0 8.5 8.0 7.5 7.0 6.5 6.0 5.5 5.0 4.5 4.0 3.5 3.0 2.5 2.0 1.5 1.0 0.5 0.0 ppm
0.91
2.00
1.00
1.07
2.92
6.06
IIIb (*cis*)

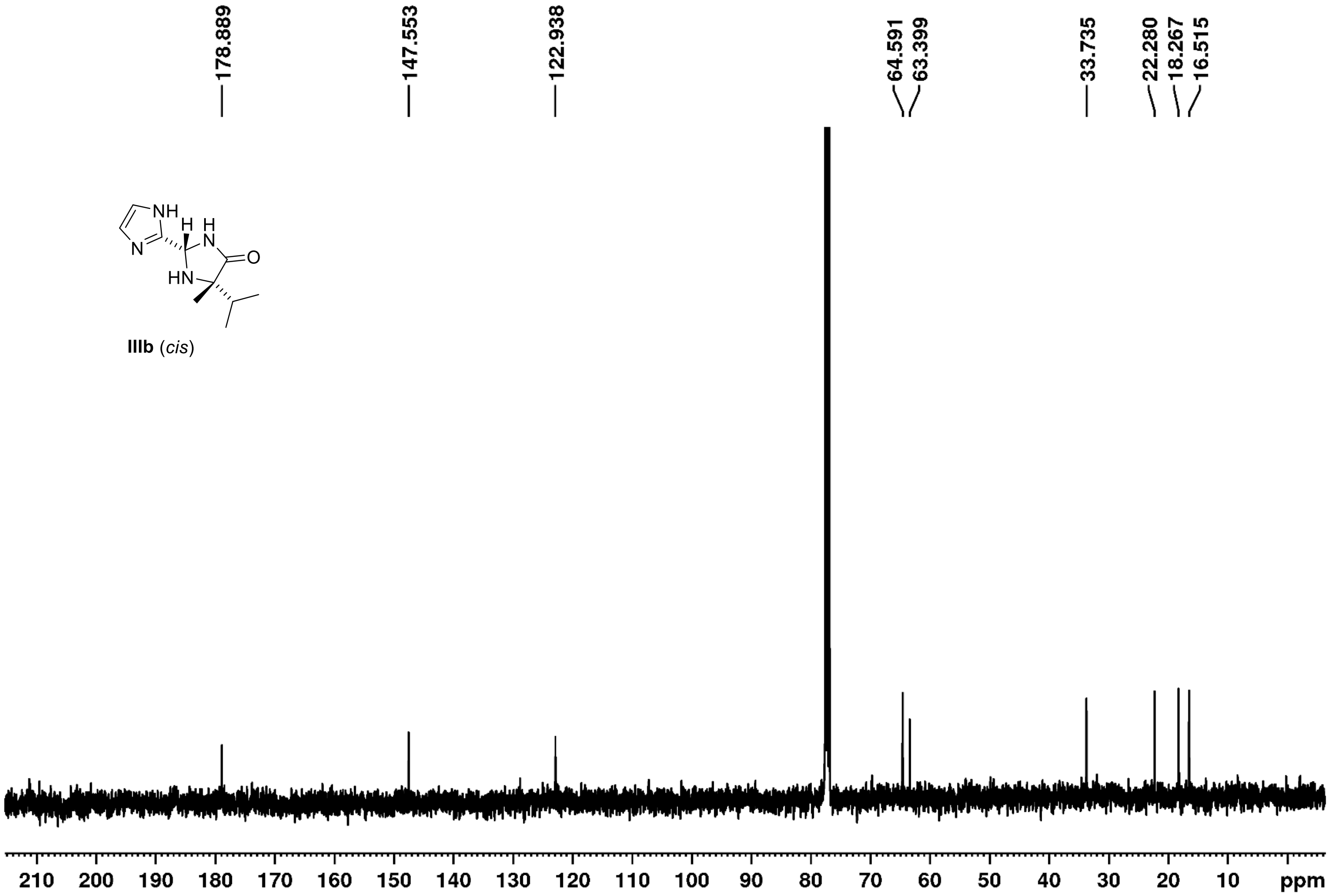
178.889
147.553
122.938
64.591
63.399
33.735
22.280
18.267
16.515
NH
H
H
N
N
O
HN
210 200 190 180 170 160 150 140 130 120 110 100 90 80 70 60 50 40 30 20 10 ppm

IIIb (*cis*)

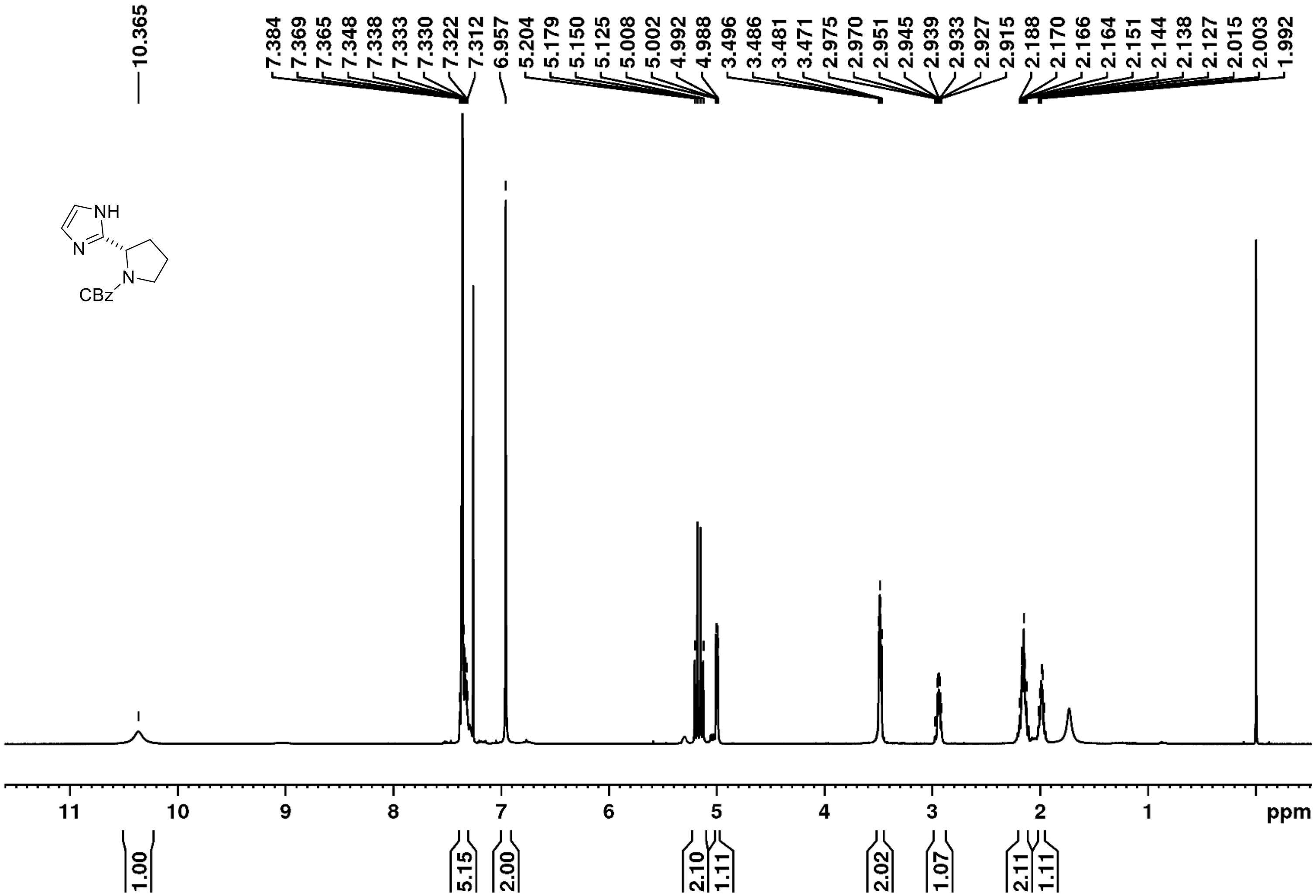
10.365
7.384
7.369
7.365
7.348
7.338
7.333
7.330
7.322
7.312
6.957
5.204
5.179
5.150
5.125
5.008
5.002
4.992
4.988
3.496
3.486
3.481
3.471
2.975
2.970
2.951
2.945
2.939
2.933
2.927
2.915
2.188
2.170
2.166
2.164
2.151
2.144
2.138
2.127
2.015
2.003
1.992
NH
N
N
CBz
11
10
9
8
7
6
5
4
3
2
1
ppm
1.00
5.15
2.00
2.10
1.11
2.02
1.07
2.11
1.11

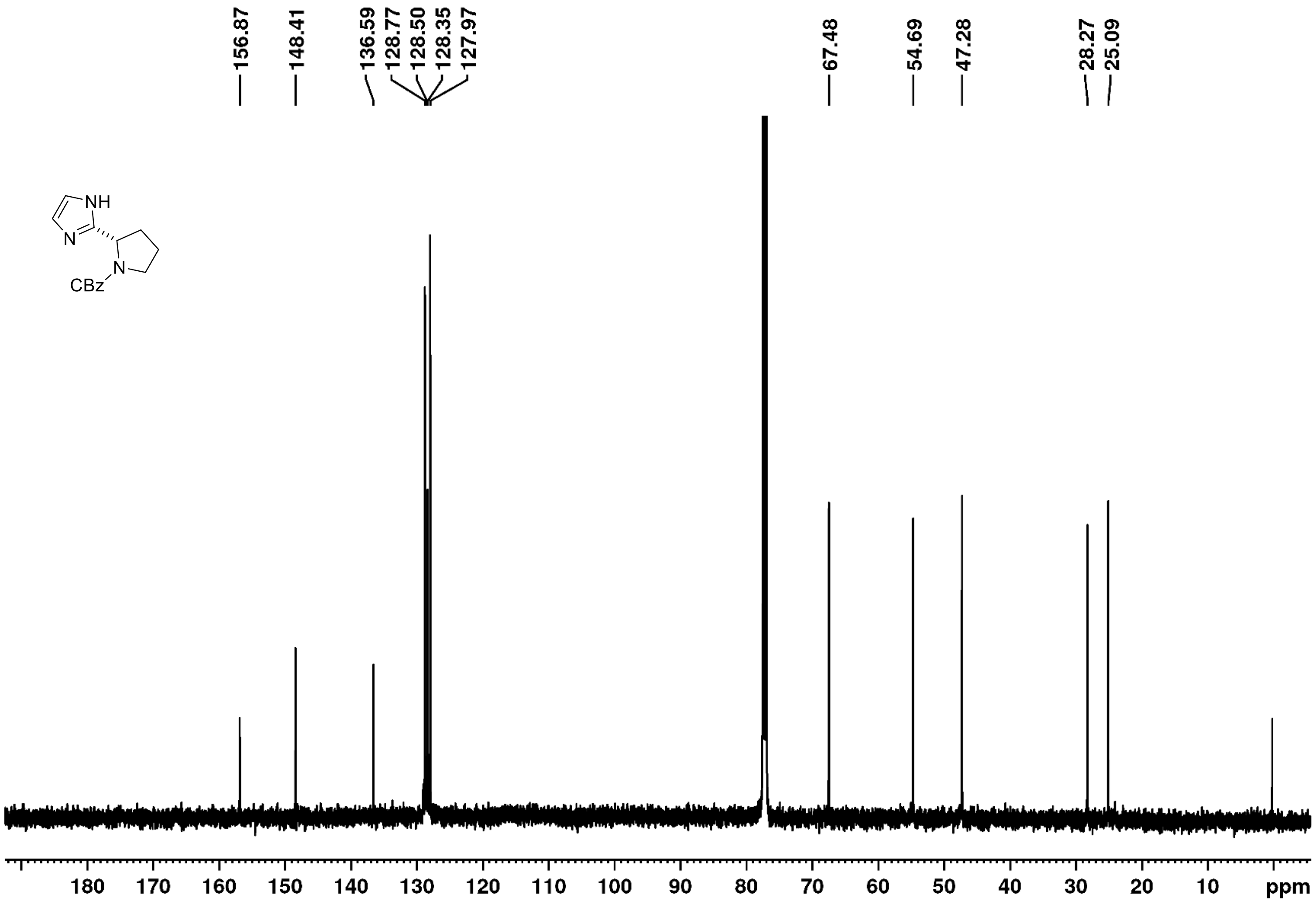
156.87
148.41
136.59
128.77
128.50
128.35
127.97
67.48
54.69
47.28
28.27
25.09
NH
N
N
CBz
180 170 160 150 140 130 120 110 100 90 80 70 60 50 40 30 20 10 ppm

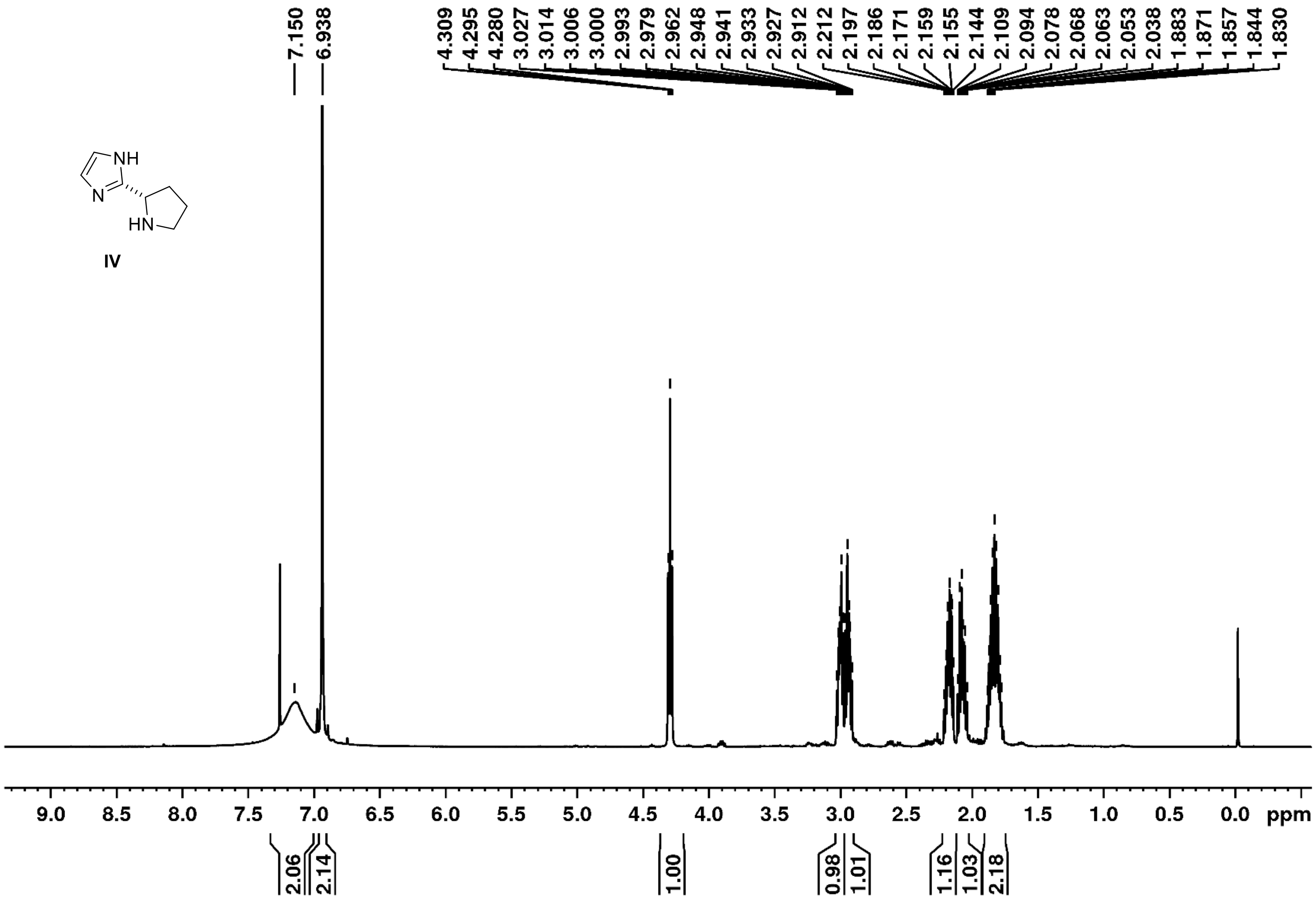

IV
7.150
6.938
4.309 4.295 4.280 3.027 3.014 3.006 3.000 2.993 2.979 2.962 2.948 2.941 2.933 2.927 2.912 2.212 2.197 2.186 2.171 2.159 2.155 2.144 2.109 2.094 2.078 2.068 2.063 2.053 2.038 1.883 1.871 1.857 1.844 1.830
9.0 8.5 8.0 7.5 7.0 6.5 6.0 5.5 5.0 4.5 4.0 3.5 3.0 2.5 2.0 1.5 1.0 0.5 0.0 ppm
2.06 2.14 1.00 0.98 1.01 1.16 1.03 2.18

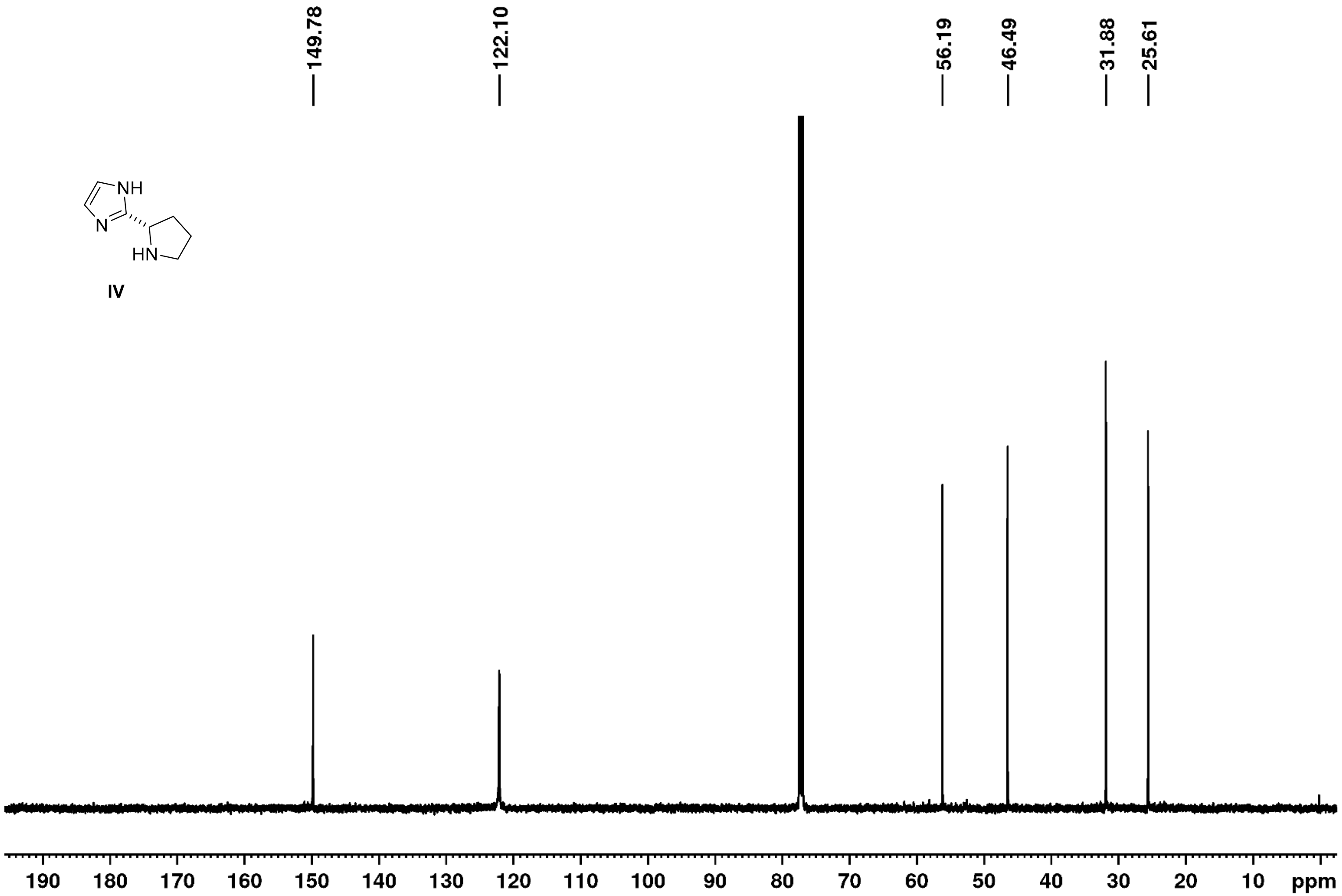
149.78
122.10
56.19
46.49
31.88
25.61
NH
N
HN
IV
190 180 170 160 150 140 130 120 110 100 90 80 70 60 50 40 30 20 10 ppm

^{1}H NMR 1D NOESY experiments:

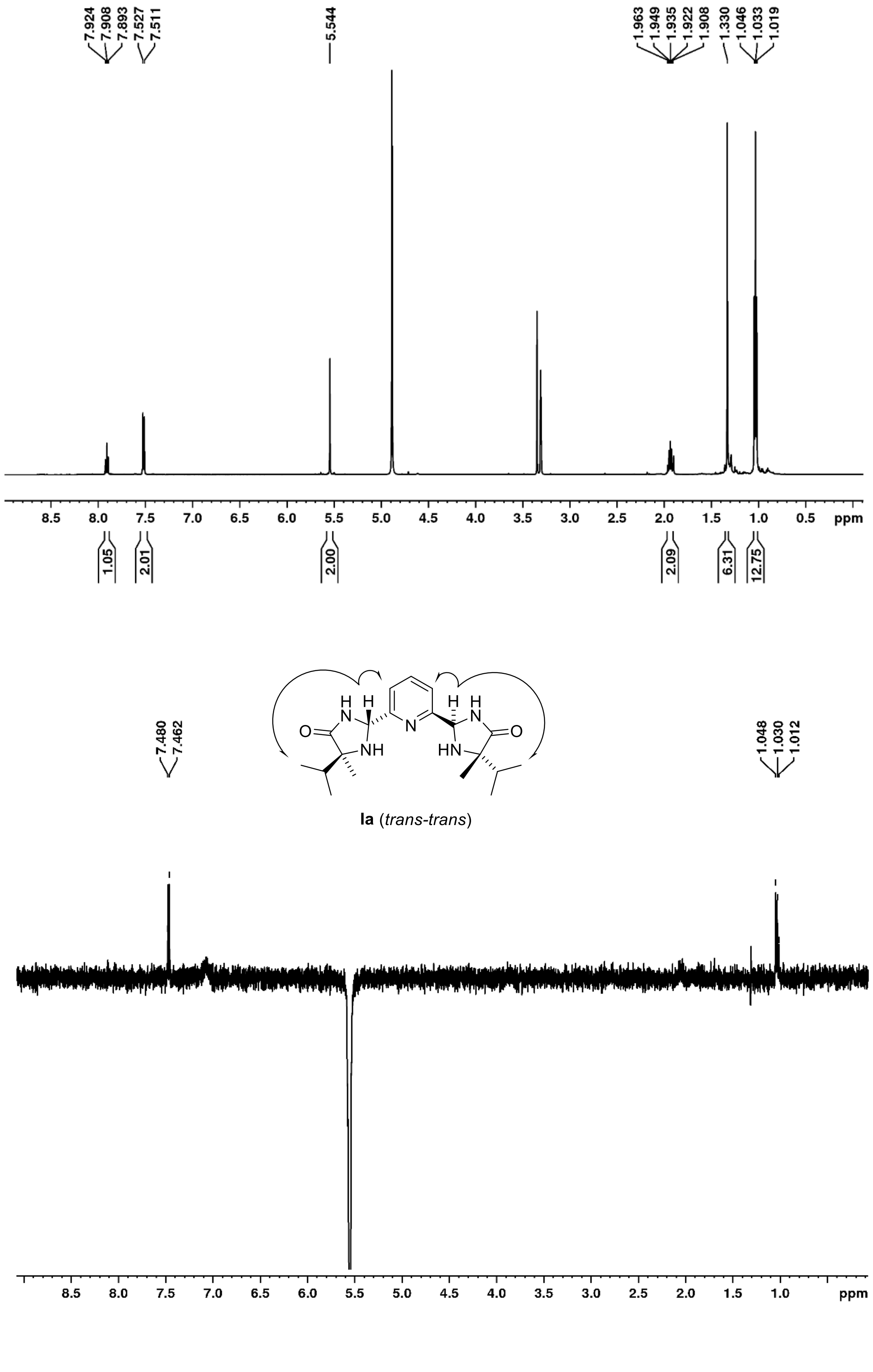

Ia (*trans-trans*)

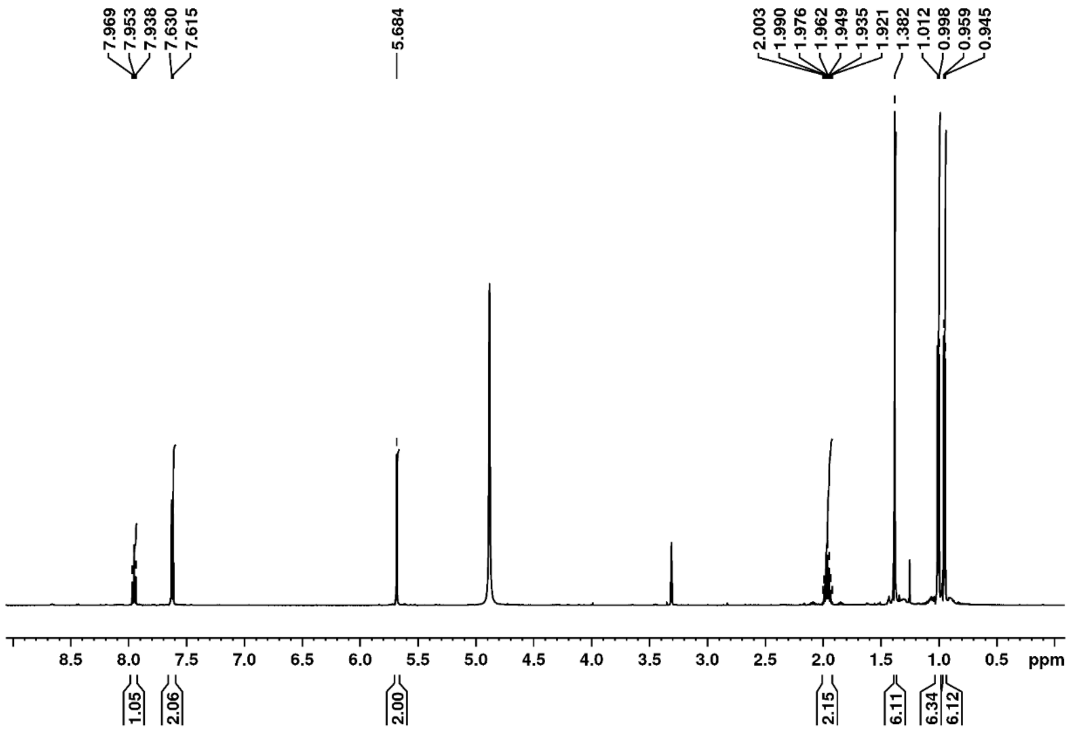
7.969
7.953
7.938
7.630
7.615
5.684
2.003
1.990
1.976
1.962
1.949
1.935
1.921
1.382
1.012
0.998
0.959
0.945
8.5 8.0 7.5 7.0 6.5 6.0 5.5 5.0 4.5 4.0 3.5 3.0 2.5 2.0 1.5 1.0 0.5 ppm
1.05
2.06
2.00
2.15
6.11
6.34
6.12

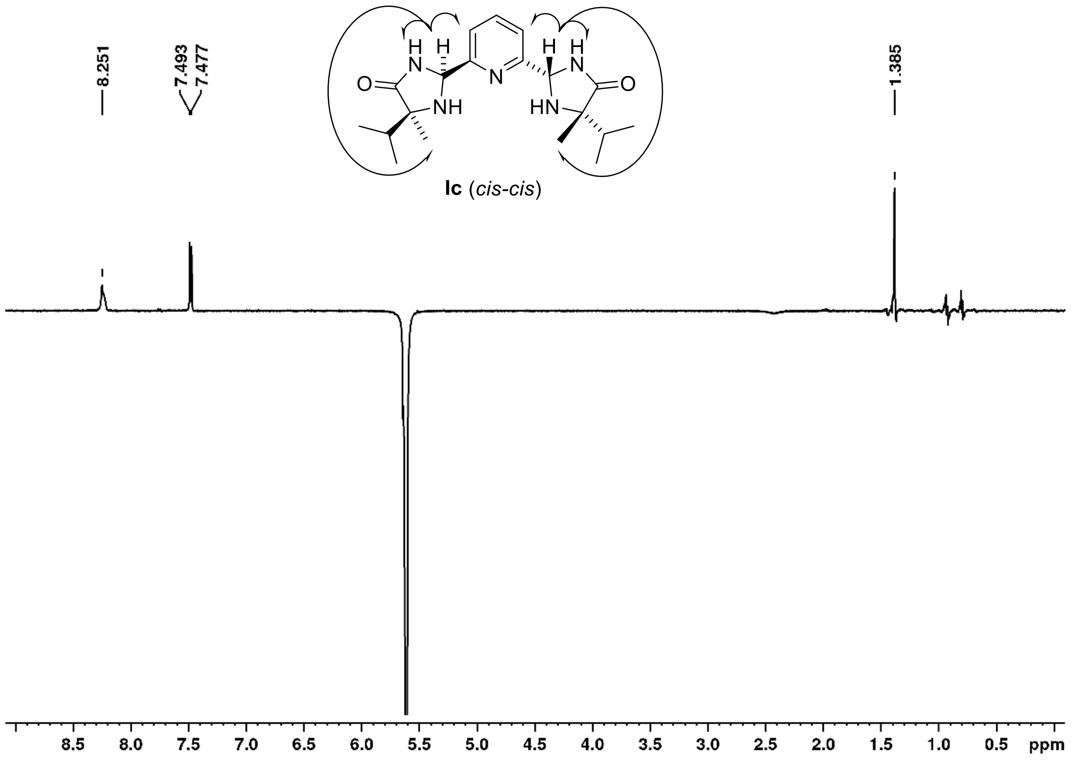
8.251
7.493
7.477
1.385
H
H
N
O
NH
N
HN
Ic (cis-cis)
8.5 8.0 7.5 7.0 6.5 6.0 5.5 5.0 4.5 4.0 3.5 3.0 2.5 2.0 1.5 1.0 0.5 ppm

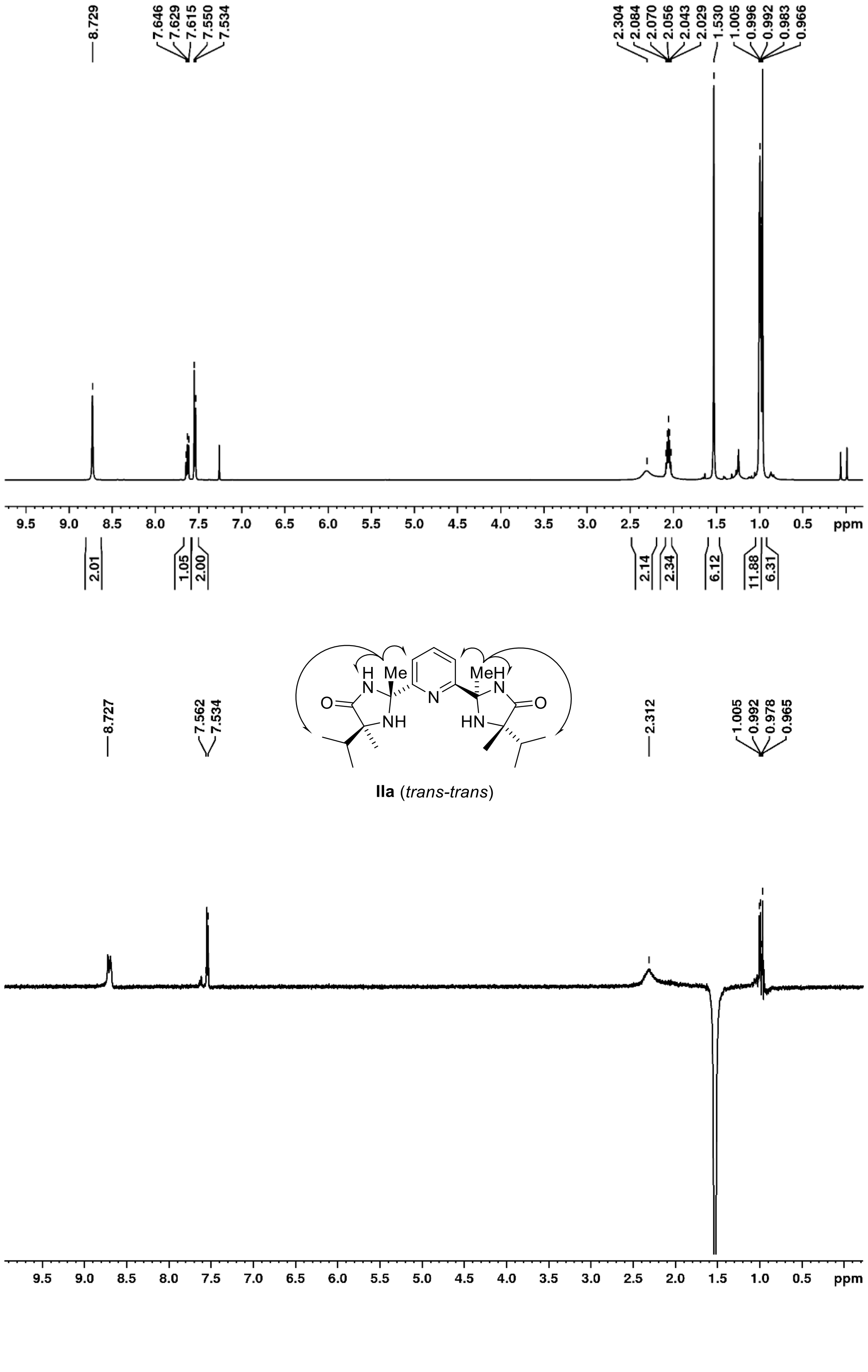

IIa (*trans-trans*)

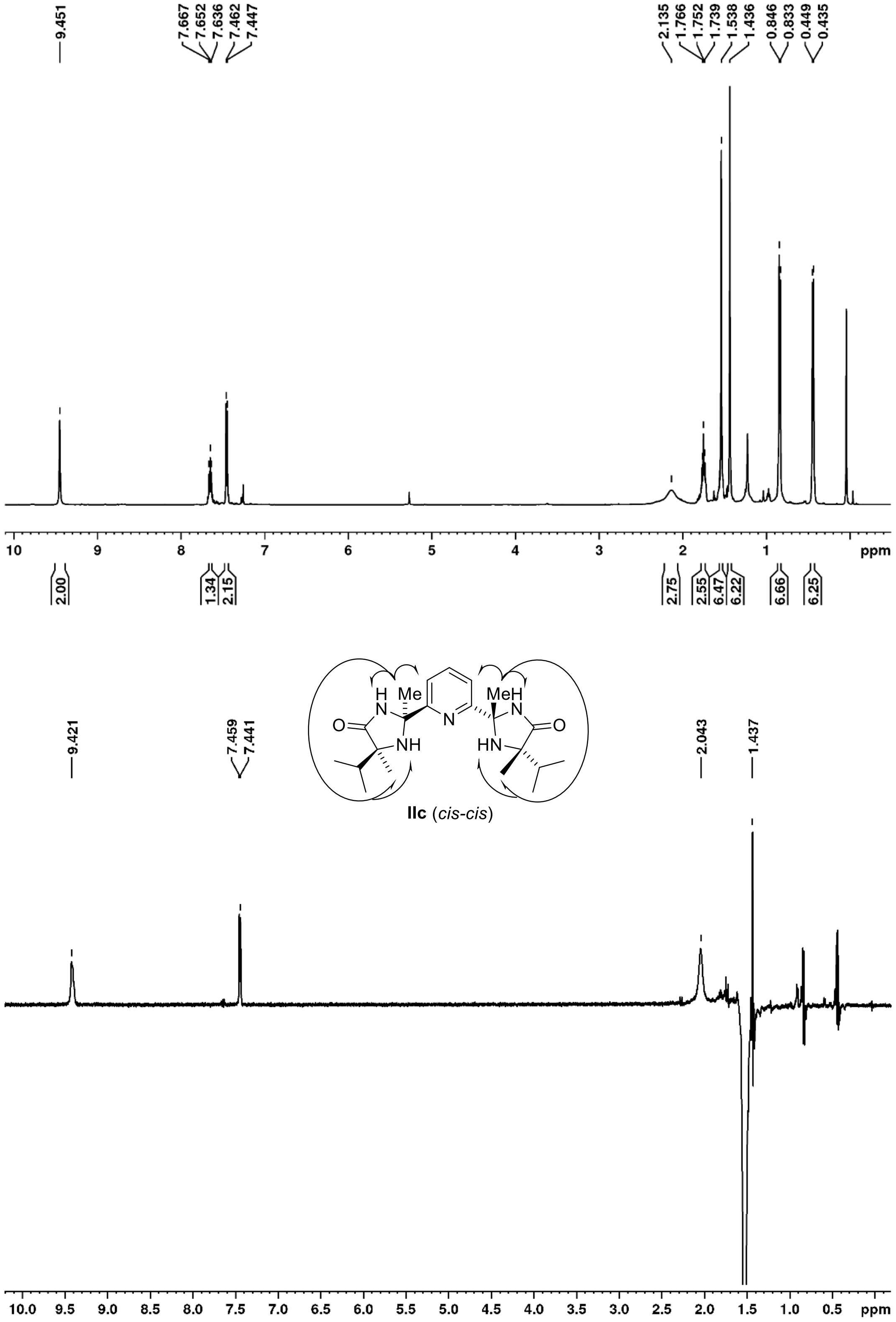
9.451
7.667
7.652
7.636
7.462
7.447
2.135
1.766
1.752
1.739
1.538
1.436
0.846
0.833
0.449
0.435
10 9 8 7 6 5 4 3 2 1 ppm
2.00
1.34
2.15
2.75
2.55
6.47
6.22
6.66
6.25
9.421
7.459
7.441
2.043
1.437
H
N
Me
MeH
N
O
NH
HN
IIc (cis-cis)
10.0 9.5 9.0 8.5 8.0 7.5 7.0 6.5 6.0 5.5 5.0 4.5 4.0 3.5 3.0 2.5 2.0 1.5 1.0 0.5 ppm

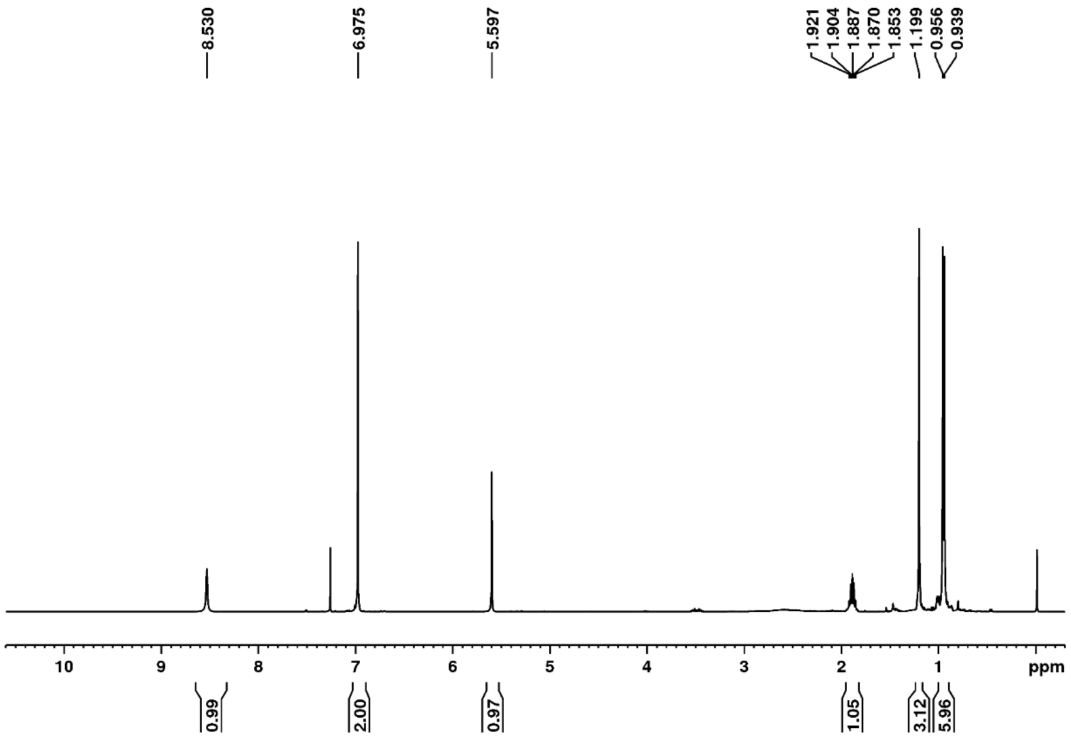
8.530
6.975
5.597
1.921
1.904
1.887
1.870
1.853
1.199
0.956
0.939
10 9 8 7 6 5 4 3 2 1 ppm
0.99
2.00
0.97
1.05
3.12
5.96

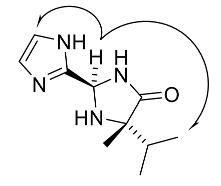
NH
H
H
N
N
O
HN

IIIa (*trans*)

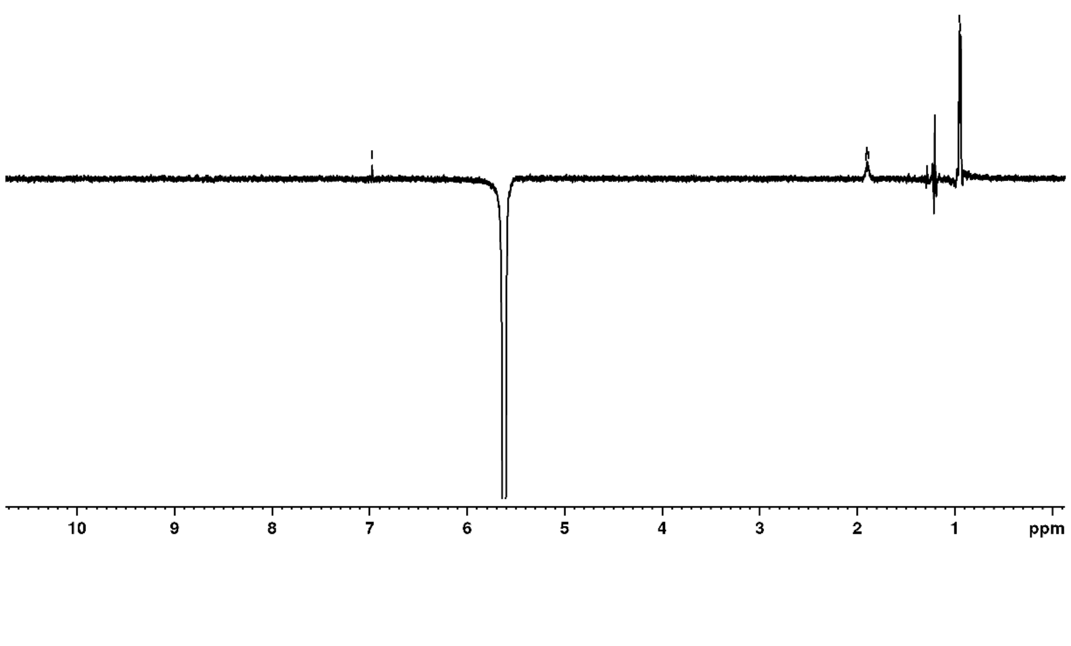
6.977
1.914
1.901
1.889
0.955
0.943
10 9 8 7 6 5 4 3 2 1 ppm

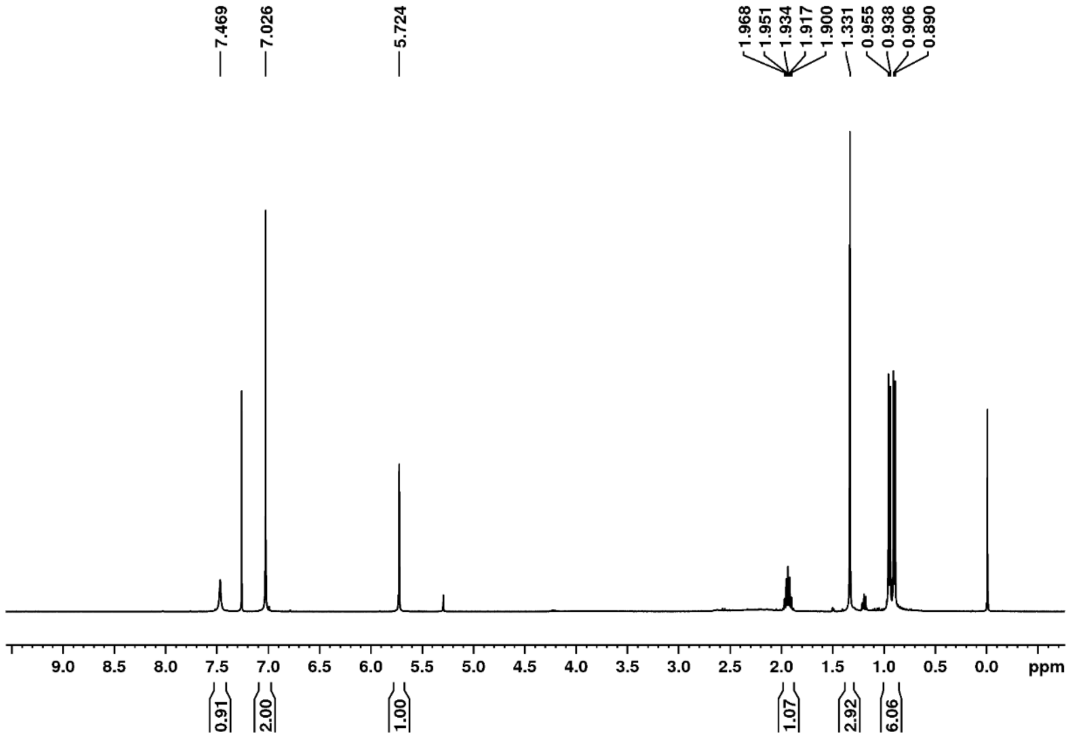
7.469
7.026
5.724
1.968
1.951
1.934
1.917
1.900
1.331
0.955
0.938
0.906
0.890
9.0 8.5 8.0 7.5 7.0 6.5 6.0 5.5 5.0 4.5 4.0 3.5 3.0 2.5 2.0 1.5 1.0 0.5 0.0 ppm
0.91
2.00
1.00
1.07
2.92
6.06

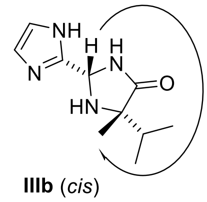
NH
H
H
N
N
O
HN

IIIb (*cis*)

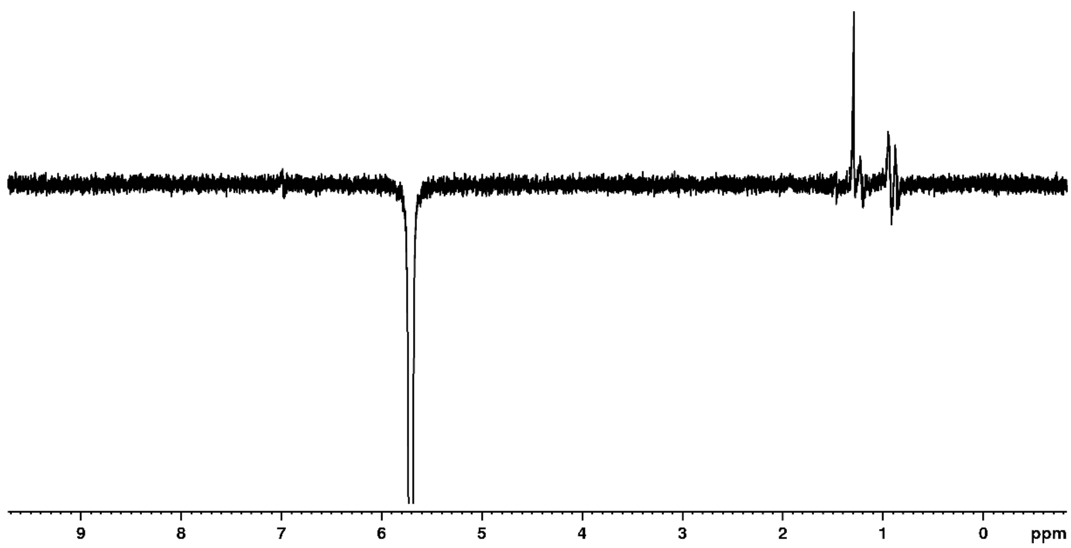
1.332
9 8 7 6 5 4 3 2 1 0 ppm

4. DFT calculations

Detailed DFT calculations were employed to analyse the structural and electronic characteristics of three nitrogen-rich molecules. The calculation was performed using Orca 5.0.2 [9,10]. The geometry of individual structures was optimised at the B3LYP/def2-TZVP level of theory with Grimme's dispersion correction D3. The optimised geometries, which do not contain imaginary frequencies, are listed below. We modelled the following compounds: Substance A (5,5-dimethyl-2-(pyridine-2-yl)imidazolidine-4-one), Substance B (2-(1*H*-imidazole-2-yl)-5,5-dimethylimidazolidine-4-one), and Substance C (2-(pyrrolidine-2-yl)-1*H*-imidazole). The primary objective of this modelling was to investigate the impact of substituting the pyridine unit with an imidazole group on the coordination potential of these molecules. A critical aspect of our study focused on comparing the Mulliken atomic charges of the coordinating nitrogen atoms.

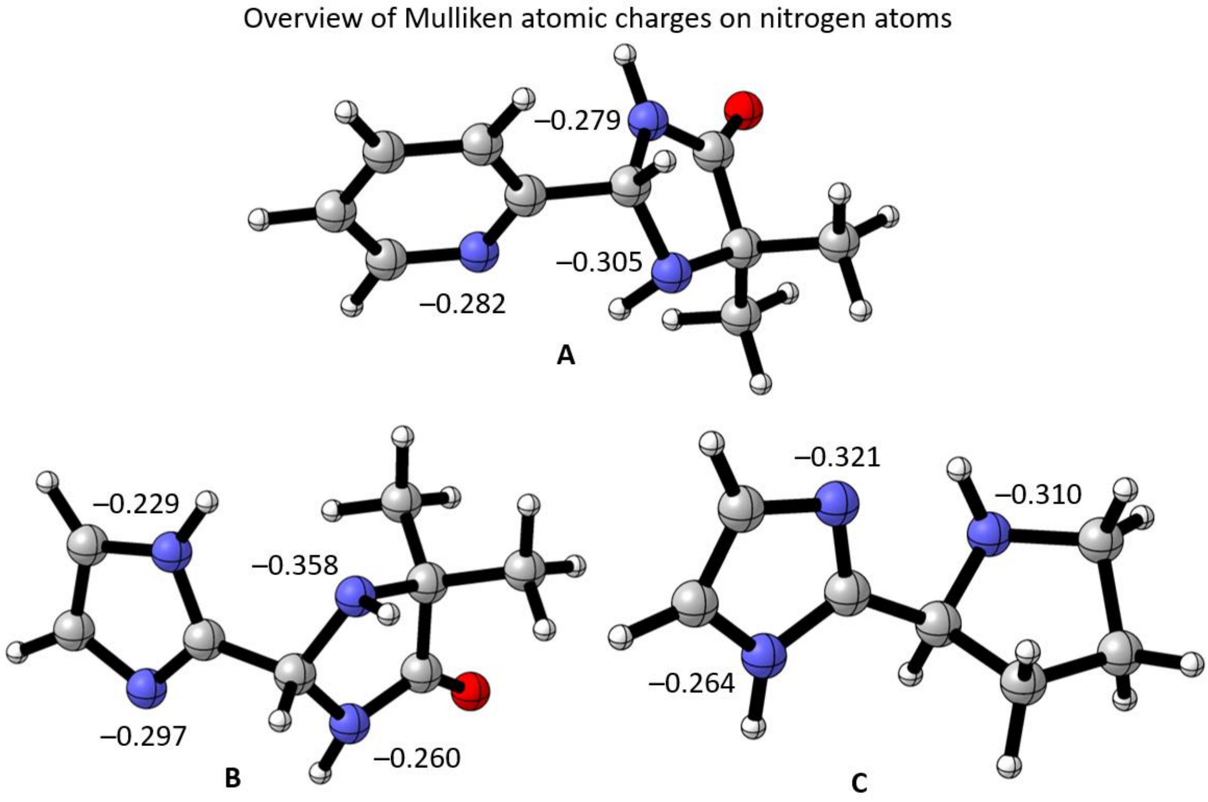

In Substance A, the pyridine nitrogen exhibited a Mulliken charge of –0.282. Intriguingly, in Substance B, where the pyridine is replaced by an imidazole ring, the corresponding nitrogen showed a higher charge of –0.297. This trend was further accentuated in Substance C, with the imidazole nitrogen displaying a charge of –0.321. These findings compellingly suggest that the replacement of pyridine with imidazole leads to an increased negative charge on the coordinating nitrogen, likely due to electron donation from the adjacent imidazole nitrogen. Consequently, our DFT analysis strongly supports the hypothesis that such a substitution would result in compounds exhibiting coordination properties closely analogous to their pyridine-based counterparts.

Optimised geometries of model compounds (in cartesian coordinates)

A – 5,5-dimethyl-2-(pyridine-2-yl)imidazolidine-4-one

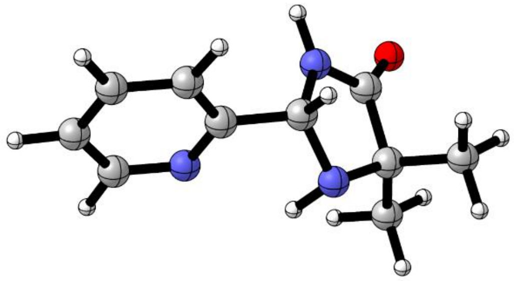

C	2.20699	–1.71209	–0.32207
C	2.58477	–2.90607	–0.92625
C	3.09198	–1.11438	0.56393
C	4.31421	–1.72839	0.80201
C	4.61087	–2.91357	0.13716
N	3.75823	–3.49387	–0.70701
H	1.91622	–3.41173	–1.61469
H	1.24235	–1.27260	–0.53721
H	2.83391	–0.19004	1.06475
H	5.02793	–1.30113	1.49494
C	5.95772	–3.58394	0.31828
N	5.86761	–5.03606	0.26144
N	6.87857	–3.24292	–0.76819
H	6.38098	–3.26083	1.27726
C	7.58688	–4.31255	–1.22177
C	7.08035	–5.52937	–0.42529
O	8.46081	–4.29954	–2.06318
H	5.05420	–5.24951	–0.30858
C	6.75795	–6.69263	–1.35030
H	5.99193	–6.41223	–2.07611
H	6.39866	–7.54593	–0.77221
H	7.65182	–6.98806	–1.89864
C	8.15502	–5.91397	0.59707
H	8.35734	–5.08846	1.28240
H	9.08560	–6.17514	0.09223
H	7.81070	–6.76569	1.18475
H	7.11623	–2.30082	–1.03321

B – 2-(1*H*-imidazole-2-yl)-5,5-dimethylimidazolidine-4-one

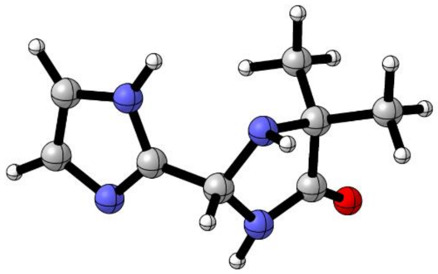

C	–0.48440	–1.37693	–0.15184
C	–1.38549	–2.55964	–0.37351
N	–1.99093	–3.02477	0.87892
C	–1.24581	–4.23081	1.32062
C	–0.45005	–4.66375	0.07507
N	–0.65282	–3.71135	–0.87942
O	0.23952	–5.65511	–0.02061
C	–0.24630	–3.88263	2.42811
N	–0.53560	–0.61742	0.97143
C	0.39586	0.38707	0.83449
C	0.96908	0.17947	–0.39172
N	0.41008	–0.92230	–0.99897
H	–2.17146	–2.27401	–1.07813
C	–2.19483	–5.33261	1.77005
H	–2.95340	–3.27773	0.70005
H	–0.01510	–3.61834	–1.65586
H	0.43161	–3.08874	2.11253
H	0.35132	–4.75812	2.68128
H	–0.78497	–3.54622	3.31568
H	–1.13523	–0.81904	1.75474
H	0.56159	1.12711	1.59702
H	1.74589	0.75367	–0.86853
H	–2.90295	–5.58863	0.97862
H	–2.75293	–5.01839	2.65450
H	–1.62651	–6.22903	2.01487

C – 2-(pyrrolidine-2-yl)-1*H*-imidazole

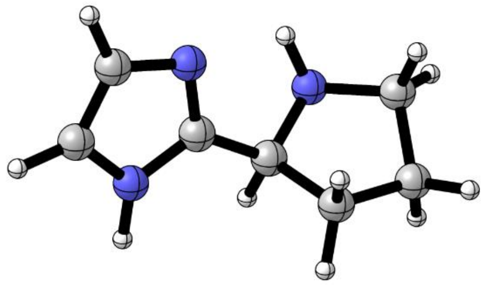

C	2.074538	–2.370055	0.844080
C	2.269428	–3.787409	1.276012
N	0.984226	–4.486498	1.414732
C	1.043107	–5.772298	0.698673
C	2.537602	–6.038418	0.502699
C	3.095959	–4.633234	0.280229
N	2.960013	–1.370482	1.119865
C	2.486871	–0.213283	0.535040
C	1.317893	–0.574643	–0.071843
N	1.072350	–1.915148	0.126633
H	2.790871	–3.778196	2.244020
H	0.244571	–3.896392	1.057367
H	3.797163	–1.458166	1.669910
H	3.007378	0.724793	0.612477
H	0.639351	0.047015	–0.631797
H	0.558097	–6.561799	1.277297
H	0.542146	–5.716853	–0.275669
H	2.965018	–6.477765	1.407848
H	2.737387	–6.717314	–0.326580
H	4.170453	–4.552477	0.450765
H	2.884199	–4.298710	–0.737956

5. Summarisation of the results of catalytic experiments

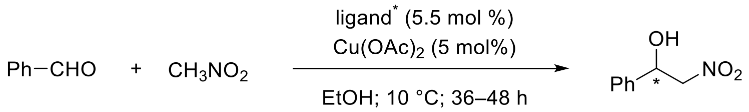

Ligand*		Time (h)	Conversion (%)[a]	ee (%)[b]	TON (–)	TOF (h^{-1})
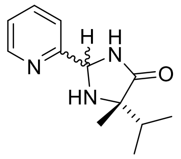 ref. [1]	(2*R*,5*S*)	36	97	92 (*R*)	18.62	0.52
	(2*S*,5*S*)	48	85	25 (*S*)	10.63	0.22
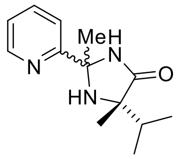 ref. [1]	(2*R*, 5*S*)	36	97	89 (*R*)	18.33	0.51
	(2*S*,5*S*)	48	80	23 (*S*)	9.84	0.21
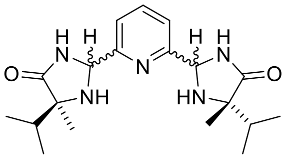	**Ia** (2*R*,2'*R*,5*S*,5'*S*)	48	99	60 (*R*)	15.84	0.33
	**Ib** (2*S*,2'*R*,5*S*,5'*S*)	48	99	38 (*R*)	13.66	0.28
	**Ic** (2*S*,2'*S*,5*S*,5'*S*)	48	99	94 (*S*)	19.21	0.40
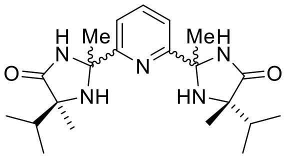	**IIa** (2*R*,2'*R*,5*S*,5'*S*)	48	99	80 (*R*)	17.82	0.37
	**IIb** (2*S*,2'*R*,5*S*,5'*S*)	48	99	70 (*R*)	16.83	0.35
	**IIc** (2*S*,2'*S*,5*S*,5'*S*)	48	99	90 (*S*)	18.81	0.39
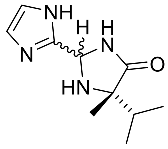	**IIIa** (2*R*,5*S*)	48	63	89 (*R*)	11.91	0.25
	**IIIb** (2*S*,5*S*)	48	57	86 (*S*)	10.60	0.22
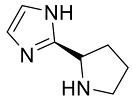	**IV** (2*S*)	48	97	50 (*S*)	14.55	0.30

[a] The conversion was determined by ^{1}H NMR analysis of the crude product.
[b] The enantiomeric excess was determined by chiral HPLC.

6. References

[1] Panov, I.; Drabina, P.; Padělková, Z.; Šimůnek, P.; Sedlák, M. *J. Org. Chem.* **2011**, *76,* 4787–4793.

[2] Moore, T. O.; Paradowski, M.; Ward, S. E. *Org. Biomol. Chem*. **2016**, *14*, 3307–3313.

[3] Zhang, B.; Jiang, Z.; Zhou, X.; Lu, S.; Li, J.; Liu, Y.; Li, C. *Angew, Chem. Int. Ed.* **2012**, *51*, 13159–13162.

[4] Wommack, A. J.; Kingsbury, J. S. *J. Org. Chem.* **2013**, *78*, 10573–10587.

[5] Wepplo, P. J. *Pestic. Sci.* **1990**, *29*, 293–315.

[6] Nováková, G.; Drabina, P.; Frumarová, B.; Sedlák, M. *Adv. Synth. Catal.* **2016**, *358,* 2541–2552.

[7] Moorthy, J. N.; Saha, S. *Eur. J. Org. Chem.* **2009**, 739–748.

[8] Mase, N.; Nakai, Y.; Ohara, N.; Yoda, H.; Takabe, K.; Tanaka, F.; Barbas C. F. III *J. Am. Chem. Soc.* **2006**, *128*, 734–735.

[9] Neese, F. The ORCA program system, *Wiley Interdiscip. Rev.: Comput. Mol. Sci.*, 2, 73-78, 2012.

[10] Neese, F. Software update: the ORCA program system, version 4.0, *Wiley Interdiscip. Rev.: Comput. Mol. Sci.*, 8, e1327, 2017.
